# Configurational heterogeneity drives songbird diversity at distinct spatial scales in managed boreal forests

**DOI:** 10.1007/s10980-026-02341-y

**Published:** 2026-03-27

**Authors:** Isabelle Lebeuf-Taylor, Juan Andrés Martínez Lanfranco, Erin Bayne

**Affiliations:** Department of Biological Sciences, CCIS 1-259, University of Alberta, Edmonton, AB Canada

**Keywords:** Multi-scale, Heterogeneity, Species diversity, Patch mosaic, Boreal forest, Songbirds

## Abstract

**Context:**

Parsing scale-dependent responses of wildlife communities to mosaic landscapes is key to evaluating conservation outcomes of natural resource development.

**Objectives:**

We evaluate how spatial heterogeneity of harvest plans and oil and gas activity influence local boreal songbird species richness at ecologically relevant spatial extents.

**Methods:**

We implemented a multi-scale framework by varying extents at which species richness and landscape configuration metrics are measured. Birds were surveyed with Passive Acoustic Monitoring (PAM) in harvest plots across forested zones of Alberta, Canada, and species richness counted at 13 increasingly broad extents. For five configuration metrics, multiple landscape extents were compared to identify scale of effects unique to each response extent.

**Results:**

Four of five metrics converged on a domain boundary at 11–14 ha. Below this threshold, within-harvest heterogeneity indexing linear features and residual retention—edge density and Shannon diversity—drove species richness. Above it, mosaic configuration metrics (patch size variation, shape complexity) became influential alongside edge density and Shannon diversity. No single landscape extent was associated with species richness across response extents.

**Conclusions:**

Effects of landscape heterogeneity on songbird diversity at within-harvest and mosaic scales can be detected from individual PAM surveys when survey extent is appropriately constrained. Empirically identifying the spatial extents at which community diversity responds to configuration allows forest planners to match monitoring design and management actions to the scale at which they will have the greatest effect.

**Supplementary Information:**

The online version contains supplementary material available at 10.1007/s10980-026-02341-y.

## Introduction

Boreal songbird communities of North America evolved in landscapes marked by frequent stand-altering disturbances. Wildfires, insect outbreaks, and wind storms, along with climate and topography, shape landscape heterogeneity in natural boreal systems (Shorohova et al. 2011; Rogers et al. 2015). However, in managed boreal forests of western Canada, patch composition and configuration are increasingly driven by natural resource development, generating mosaics that differ markedly from the historical range of variability (Cyr et al. 2009; Pickell et al. 2015). Industrial disturbances—including timber harvest, seismic lines, pipelines, and wellsites—create novel spatial patterns characterized by high edge density, atypically straight patch edges, reduced contiguity and amount of old growth forests, and homogenous patch shapes (Kuuluvainen and Gauthier 2018; Riva and Nielsen 2020). Boreal songbirds are responsive to the compositional and configurational heterogeneity of these mosaic landscapes (Leonard et al. 2008; Kroll et al. 2017; Crosby et al. 2019), which retain varying levels of broadleaf, mixedwood, and coniferous forests, shrubby openings, and successional stages (Schieck et al. 1995; Hobson and Schieck 1999).

Landscape heterogeneity—which includes compositional diversity (variety of land-cover types) and configurational diversity (spatial arrangement and shapes of patches; Tonetti et al. 2023)—drives songbird species diversity through multiple mechanisms (Dunning et al. 1992; Tscharntke et al. 2012). Local communities are assembled through environmental filtering, where colonization, competition, and extinction dynamics are contingent on species movement through connected or isolated patches (Gustafson and Gardner 1996), availability of key resources, competitive interactions between resource generalists and strong colonizers (Zhang et al. 2024), and local habitat loss (Kadmon and Allouche 2007). Configuration influences these assembly processes via dispersal, edge effects, and access to complementary resources across patch boundaries. Maintaining structural and compositional heterogeneity in managed forests tends to benefit songbird species (Fiss et al. 2025), but the parameters of this heterogeneity are difficult to generalize. At the local level, increased configurational heterogeneity generates higher densities and variety of resources, potentially supporting greater species richness (Zhang et al. 2013). However, increasing heterogeneity may reduce the amount of specific resource types below thresholds necessary to support viable populations of some species (Mahon et al. 2019). Non-linear relationships between heterogeneity and diversity are well documented (Tscharntke et al. 2012; Zhang et al. 2024), where benefits of resource diversification may be offset by reductions in key resource availability beyond some threshold level of landscape complexity (Allouche et al. 2012).

Characterizing these complex relationships requires measuring heterogeneity with multiple metrics that capture distinct aspects of configurational diversity at multiple scales, rather than relying on single indices that may miss ecologically relevant patterns (Jokimäki and Huhta 1996; McGarigal et al. 2016). In multi-stressor landscapes like the boreal forest of western Canada, forest management practices generate a wide range of landscape complexities, in part due to evolving forestry practices shifting toward natural disturbance emulation and the uneven spatial distribution of exploitable resources across regions (Groot et al. 2005; Johnstone et al. 2016). Concerns about configurational patterns created by industrial forestry led to shifts from repeated simple patch geometries to a variety of polygonal shapes, complex edges, within-harvest plot retention of mature trees, and retention of large remnant forest patches for higher connectivity (Kuuluvainen and Grenfell 2012; Venier et al. 2014; Stanturf 2015).

Just as species experience their surroundings and respond to patch configuration at different spatial scales, community responses to heterogeneity are also scale-dependent (Wiens 1989; Levin 1992; Jokimäki and Huhta 1996; Tscharntke et al. 2012). Key to elucidating this scale dependence is in varying the spatial scale in which response and predictor variables are measured (McGarigal et al. 2016; Crosby et al. 2023). Scale consists of the extent and grain at which spatial objects or processes occur temporally and spatially, with profound implications for accurately gauging species response to changing environments (Turner 1989; Gustafson 1998). The arbitrary selection of spatial measurement extents in heterogeneity-diversity studies often leads to seemingly contradictory or inconsistent results (Tamme et al. 2010; McGarigal et al. 2016; Cours and Duflot 2025), which hinders our ability to extrapolate findings between scales or geographic contexts. Since environmental variables vary along different scale continua and species respond depending on their ecology, there are two critical components to selecting spatial extents: quantifying environmental variables at the scales at which they affect habitat suitability for species, and measuring corresponding changes in ecological communities at the scales these changes occur (Wheatley and Johnson 2009). Community assembly emerges from scale-dependent population-level dynamics that do not occur at any single spatial extent (Jokimäki and Huhta 1996; Zhang et al. 2024), requiring analyses that systematically vary both response and landscape extents.

In habitat association studies in heterogeneous environments, the role of landscape extent has been well documented (eg: Holland et al. 2004; Juárez–Fragoso et al. 2024; Branney et al. 2024). Typically, predictor variables (e.g., landscape configuration metrics) are measured and compared across multiple spatial extents to identify a ‘scale of effect’–the spatial extent where effects are strongest, and therefore assumed to be more ecologically relevant (Vernier 1995; Jackson and Fahrig 2012; McGarigal et al. 2016). The role of response extent is equally important, where it is mostly handled in two ways in passive acoustic monitoring: by fixing the detection radius of point counts into discrete buffers (Hutto et al. 1986), and by nesting or pooling local surveys into larger units of replication (Mahon et al. 2016, 2019; Crosby et al. 2023; Fiss et al. 2025). For example, in the boreal region of northern Alberta, Canada, the strength of association between abundance and energy sector disturbances varied with area surveyed at the survey level, where birds were counted within 50 m, 100 m or unlimited distance human point counts (Bayne et al. 2016). Although varying response extents at the local survey level is common for point counts (Hutto et al. 1986), it is scarcely implemented in passive acoustic monitoring, stemming from the difficulty of estimating recording unit detection area (except see Darras et al. 2016, 2018; Hedley et al. 2021; Lebeuf-Taylor et al. 2025).

Recently, several tools to estimate distance of vocalising birds from PAM recording units have been developed, including localization arrays (Efford et al. 2009; Rhinehart et al. 2020), recognizer scores (Knight and Bayne 2018; Pérez-Granados 2023), and excess attenuation regression models, which partition sound attenuation between physics-based and excess sources (Yip et al. 2017; Hedley et al. 2021; Haupert et al. 2023). These models predict distance from song amplitude and forest characteristics, so vocalizations can be truncated at select distances to create multiple detection areas around individual Autonomous Recording Units (ARUs), yielding nested measures of species richness at different spatial extents (Lebeuf-Taylor et al. 2025). To our knowledge, comparing multiple response extents has not been applied in diversity–disturbance associations, and has the potential to reveal scale-dependence in both the response and predictor variables. This method enables identification of scales of effect—spatial extents where species respond most strongly to environmental variation—and domains of scale—spatial ranges where spatial relationships between response and predictor remain consistent (Wheatley 2010)—from surveys with single ARUs.

Because PAM studies have not addressed scale dependence in response variables at the local survey level, the implication of arbitrarily selecting response variable extents on landscape management are unknown. While numerous studies carefully examine scale-dependent effects of landscape heterogeneity on species diversity at broad spatial extents or across large scale contrasts (e.g., local vs. regional), scale optimization within local landscapes remains understudied. Here, we use songbird species richness in harvested areas in the boreal and hemiboreal forests of Alberta, Canada, to see how spatial heterogeneity of harvest plans influences outcomes via scale. Our objectives were to (1) assess whether there were identifiable extents within which species richness is more strongly associated with configuration metrics, and (2) identify domains of scale between response and landscape extents to gauge consistency of configuration effects across spatial extents. By examining scales of effect and domains of scale, we aimed to determine whether arbitrary versus informed selection of response and landscape extents would impact interpretation of forest management effects on songbird diversity, ultimately improving our ability to predict and manage anthropogenic changes to landscape heterogeneity in dynamic boreal landscapes.

## Methods

### Study area and vegetation classification

**Fig. 1:**
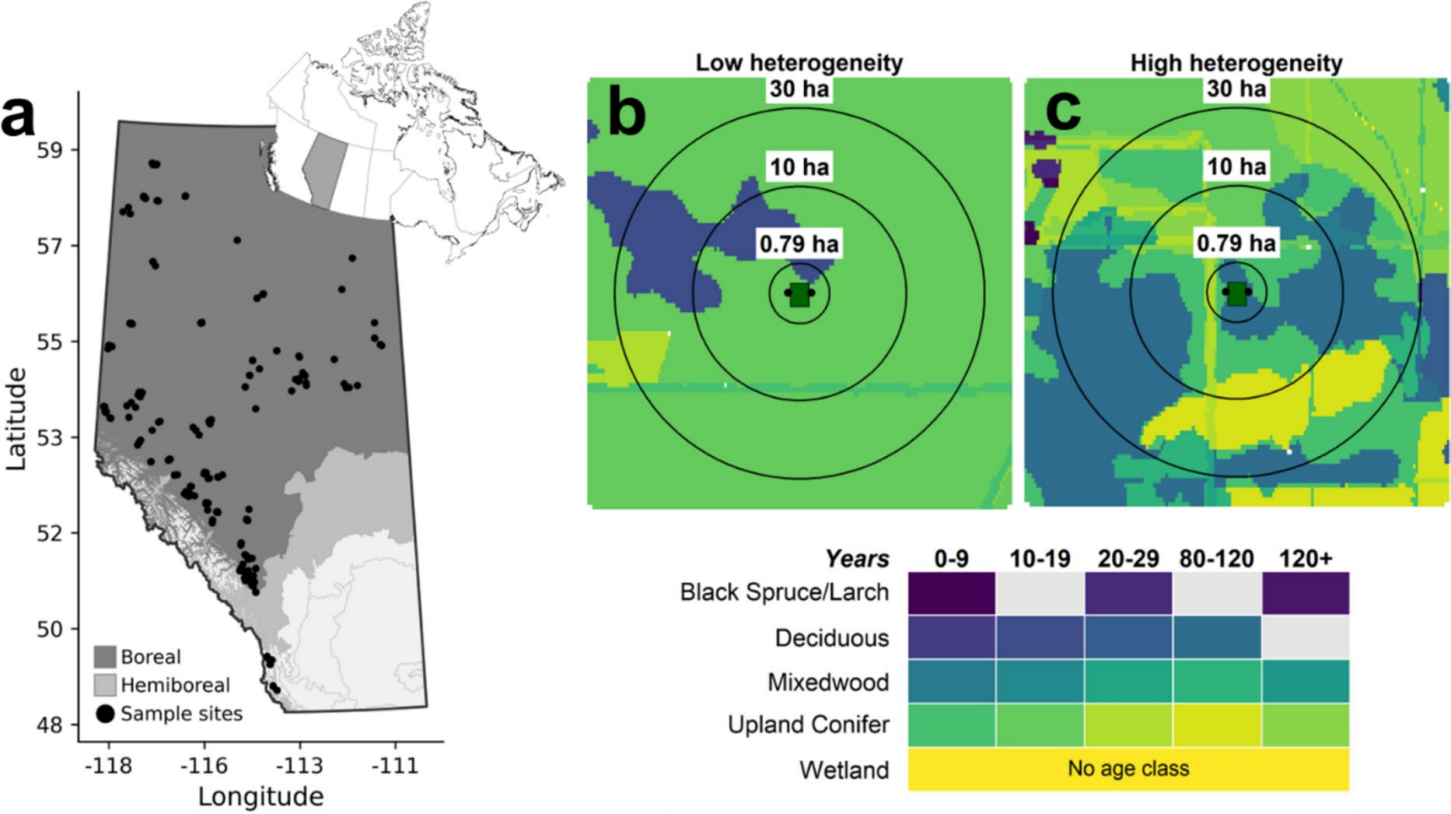
Study system in Alberta, Canada. **a** Sampling sites (n = 392) in boreal and hemiboreal natural regions; inset shows location within Canada. **b**, **c** Example landscapes representing low and high configurational heterogeneity, with landscape metrics calculated at an extent of 78.5 ha (500 m radius). Concentric circles show nested spatial extents (0.79, 10, 30 ha; 50, 178, 309 m radius) at which species richness can be measured. Land cover types are classified by dominant vegetation and age class (years since disturbance); the color legend applies to both panels

We conducted the study in primarily upland forests in the Boreal and Foothill Natural Regions of Alberta, Canada (Fig. 1), where single ARUs a minimum of 300 m apart were affixed within timber harvest areas (n = 392). Sampling locations were in 19 different Forest Management Agreements, which are regions for which specific foresters and mills hold logging rights (Government of Alberta 2023). Harvest areas ranged from 7 ha upward, with a mean of 11 ha (187 m radius). Dominant vegetation within 80 hectares of harvest areas mostly consist of deciduous stands of *Populus tremuloides* (aspen), *P. balsamifera* (balsam poplar) and *Betula papyrifera* (white birch), pure stands of *Pinus banksiana* (jack pine) or *P. contorta* (lodgepole pine), conifer stands of *Picea glauca* (white spruce) or *P. engelmannii* (Engelmann spruce), mixedwood stands of upland spruce and deciduous trees, upland shrubs, grass, and forbs, and lowland stands of *P. mariana* (black spruce), *Larix laricina* (tamarack), or untreed bogs and fens. Stand composition, age, and disturbance origin were extracted from the Alberta Vegetation and Human Footprint Inventories polygonal geodatabase (ABMI 2024). The inventories outline polygons and polylines of each cover type, their year of origin and type of disturbance, if pertinent. We rasterized the polygonal geodatabase to a 5 m grain with the R package *terra* (Hijmans 2024). This grain excludes both streams and narrow seismic lines, which tend not to impact birds at the community level (Machtans 2006), but is fine enough to capture spatial and age heterogeneity in harvest areas and wide linear features, which both influence community composition of birds (Gustafsson et al. 2012; Kalukapuge et al. 2024; Lebeuf-Taylor et al. 2025).

### Broad vegetation classes

Cover types were categorized by dominant vegetation and age class, where forests were binned into 10 year increments in their first 80 years of regeneration, followed by 80–119 and 120 + years to represent mature and old growth conditions. Untreed vegetated uplands were consolidated into a single grass and shrub category, capturing open canopy conditions. Lowlands were broadly categorized as forests or non-forested wetlands. Vegetated lowlands in our study provide habitat for some species in the boreal bird community, but are not usually disturbed by timber harvesting. Permanent disturbances such as roads and gravel pits, while overall uncommon around study sites, were treated as empty cells in the rasters.

To characterise landscape heterogeneity, we calculated five indices at the ‘landscape-level’ (in the configuration metrics sense; see FragStats documentation to differentiate patch, class, and landscape level (McGarigal et al. 2023)): edge density (m/ha), Shannon diversity index (SHDI), perimeter-area fractal dimension (PAFRAC), core area index coefficient of variation (%) (CAI CV), and patch contagion (CONTAG). Metrics were extracted with the *landscapemetrics* package (Hesselbarth et al. 2019) in R 4.3.3 (R Core Team 2024); consult package and FRAGSTATS documentation for full details on metric calculations (McGarigal et al. 2023). Each metric was calculated within increasing buffer sizes around survey points, from 50—500 m radius (0.79—78.5 ha), in increments of 2 m increase in radius (232 extents). The minimum 50 m radius matched the finest response extent, whereas the maximum 500 m radius (78.5 ha) was selected to capture landscape context beyond the largest harvest blocks in our study area while reducing overlap between study sites.

In terms of composition, all upland cover types were treated of equal value. We focused on configuration and richness metrics. Edge density captures both the density of linear features (seismic lines, pipelines, roads) and the shape complexity of harvest patches, indexing the shift from repeated straight edges of conventional logging to irregular boundaries under natural disturbance emulation. Shannon diversity—indexing the number and abundance of uniquely vegetated patches—captures both natural landscape heterogeneity and the temporal intensity of logging, as differently-aged stands in concentrated areas generate more vegetation classes. CAI CV is an area-agnostic metric of variation in core area of patches; landscapes with checkerboard-style logging have low CAI CV, whereas landscapes with a mix of small and large patches have high CAI CV. Core area at the class level contributes to bird community structure (Mahon et al. 2016), especially for sensitive interior forest species (Austen et al. 2001). PAFRAC indexes patch shape complexity, contrasting simple geometric shapes with complex, irregular boundaries. CONTAG measures aggregation, where higher values indicate large, continuous patches with less intermixing of vegetation classes; the spatial subdivision of large, contiguous mature forests is associated with declines of sensitive mature forest species (Schmiegelow et al. 1997; Hart et al. 2025).

### Acoustic surveys and measuring species richness

Acoustic surveys were conducted between 2014 and 2023. We deployed one ARU per site, affixed ~ 1–1.5 m from the ground and facing north, randomly placed within harvested areas in the first two decades of regeneration — a period of rapid vegetation structural change and bird community turnover (Schieck and Song 2006). ARUs were a minimum of 300 m from one another (median nearest-neighbour distance = 430 m) and from roads, and a minimum of 150 m from harvest edges. ARUs recorded 3-min segments every 20 min during the first four hours from sunrise during the migratory bird breeding season (May 25 to July 6). At each site, we selected a single recording without audible wind, rain, or industrial noise that would mask bird vocalizations. From this recording, the loudest and least masked song of each passerine species was tagged in a spectrogram through the *Wildtrax* platform (https://wildtrax.ca). Detections were filtered out if they were quieter than the reference amplitude for that species, given site condition and the selected truncation distance. To calculate species richness within specific areas, we truncated the distance at which birds are counted in increasing distance increments from the ARU, from 50 m radius to 350 m every 25 m (0.79 to 38 ha), where 350 m reaches the maximum detection distance for many songbirds with modern ARUs (Darras et al. 2018), but some species in open canopies, like the Olive-sided Flycatcher (*Contopus cooperi*) will still be detected (Yip et al. 2017); see Lebeuf-Taylor et al. (2025) for a full description of truncation and distance estimation method. Species richness was limited to passerines as the distance truncation method has only been calibrated for North American boreal passerine vocalizations.

### Statistical analysis

The global model structure was

$$\begin{aligned} {\mathrm{Log}}\,\,({\mathrm{E}}\left[ {{\text{Species richness}}_{{{\mathrm{extent}}i}} } \right] & = {\mathrm{intercept}} + \beta_{{{\mathrm{metric}}\,\,{\mathrm{at}}\,\,{\mathrm{extent}}\,\,j}} *{\mathrm{configuration}}\,\,\,{\mathrm{metric}}_{{{\mathrm{extent}}\,\,j}} + \beta_{{{\mathrm{upland}}\,\,{\mathrm{s}}}} *{\mathrm{proportion}}\,\,{\mathrm{uplands}}_{{{\mathrm{extent}}\,\,j}} \\ & \quad + \beta_{{{\mathrm{GDD}}}} *{\mathrm{GDD}} + \beta_{{{\mathrm{autocov}}}} *{\mathrm{spatial}}\,\,{\mathrm{autocovariate}} + \alpha_{{{\mathrm{survey}}\,\,{\mathrm{year}}}} \\ & \quad \alpha_{{{\mathrm{survey}}\,\,{\mathrm{year}}}} \sim Normal\left( {0, \, \sigma^{{2}}_{\alpha } } \right) \\ \end{aligned}$$

where *i* is from 50 to 350 m radius in increments of 25 m (13 extents), and *j* is from *i* to 500 m radius, in 2 m increments. We included proportion upland, which mostly varied between 0.8 to 1.0, as a predictor to modulate local productivity as some species of boreal songbirds occupy lowlands in much lower densities (Mahon et al. 2016). Given our study area's broad latitudinal range, and the different temperature-moisture regimes between the boreal and Canadian Rocky Mountain foothills, growing degree days (GDD; days above 5 °C) accounts for top-down regional productivity effects. GDD was extracted with ENVIREM at 1-km resolution (Title 2018). The spatial autocovariate, which can be understood as a residual autocorrelation process, accounts for unmodelled spatial correlation by extracting fitted values of a spatial smooth on residuals from an initial non-spatial model. Survey year is a random effect that captures yearly variation in abundance and distributions. Models were Maximum Likelihood Estimate generalized linear models, with a negative binomial or Poisson distribution, depending on data dispersion, with *glmmTMB* in R 4.3.3 (Brooks et al. 2017; R Core Team 2024).

### Optimizing extent selection

For our first objective, we assessed scale dependence in response and predictor variables in a two-step, multiple uniscale regression framework. We refer to the spatial extent at which landscape configuration metrics were measured as the *landscape extent*, and the spatial extent at which species richness was measured as the *response extent*. First, for each response extent (13 extents, from 0.79 ha to 38 ha), we compared the strength of association with each landscape configuration metric at increasingly large extents, from 0.79 ha to 78.5 ha. The minimum extent for the landscape configuration metric matched the response extent. For example, for models where birds were measured within a 50 m radius, the smallest landscape extent was 50 m radius, so there were 232 models (one for each extent within which the configuration metric is measured). When birds were counted within a 250 m radius, the minimum landscape extent was 250 m radius, and there were 129 models. Proportion uplands was measured at the same extent as the configuration metric.

Our framework for identifying the scale of effect prioritized models where the landscape configuration metric was statistically significant (α ≤ 0.05), selecting the model with the highest marginal R^2^ from this subset. If no models met this criterion, we selected the model with the highest marginal R^2^ among those with p-values between 0.05 and 0.1. If neither condition was satisfied, we selected the model with the highest marginal R^2^ from all remaining models. Scale selection literature emphasizes biologically informed predictions linking species traits to scale selection (Jackson and Fahrig 2015). However, since community assembly processes lack the mechanistic a priori predictions available for single-species responses, we chose marginal R^2^, which quantifies the proportion of variance explained by fixed effects (Nakagawa and Schielzeth 2013), as our empirically derived criterion. Prioritizing statistical significance introduces potential for inflated Type I error rates across the multiple comparisons. However, our inference does not rest on individual models at single extents; rather, we interpret the consistency of effect direction and magnitude across response and landscape extents. A spurious result at one extent would not produce coherent cross-scale patterns, whereas an isolated statistically significant effect unsupported by consistent directionality at neighboring extents should be interpreted with skepticism.

Next, we compared three competing fixed effect structures for each response extent, keeping the configuration metric’s landscape extent at its scale of effect and proportion uplands at the same extent as the configuration metric. These were (1) additive effects of metric + proportion uplands, (2) interactive effects of metric × proportion uplands, or (3) polynomial effects of metric + metric^2^ + proportion uplands. Prior to comparing fixed effect structures, we determined whether to include a random intercept for survey year by fitting the additive model with and without the random effect and evaluating model residuals for overdispersion, spatial autocorrelation, and normality using the *DHARMa* package in R (Hartig 2022). We then held the random effect structure constant (either including or excluding the year random intercept based on this initial assessment), consistently included the spatial autocovariate across response extents, and conducted pairwise likelihood ratio tests among the three fixed effect structures (α = 0.05). When the likelihood ratio tests indicated no significant difference between nested models (P > 0.05), we selected the most parsimonious model.

### Consistency of scale dependence

To meet our second objective of identifying domains of scale, we fit a hierarchical generalized additive model (HGAM) between landscape extents and response extents to gauge the consistency of the extents where scale of effects occurred. We fit the HGAM with the *mgcv* package in R with both independent shapes and wiggliness (no global smooth), which allows inflection points to vary among configuration metrics (Wood 2017; Pedersen et al. 2019). Because site-level surveys are nested within greater extents, and we are comparing response and predictor variable associations at increasingly broad extents within the same sites, observations are linked through spatial autocorrelation (Tobler 1970; Fletcher and Fortin 2019).

Our a priori predictions about scale domain structure were: (1) if spatial autocorrelation processes dominated, landscape extents would increase linearly with response extents (1:1 relationship) with no domain boundaries; (2) abrupt shifts in landscape extent relative to response extent would indicate domain boundaries reflecting scale-dependent relationships between response and predictor variables; and (3) within scale domains, relationships would be consistent (flat or stable slopes), indicating that the scale of effect for landscape heterogeneity remains constant within that domain (Wiens 1989; Wheatley 2010), regardless of minor changes in measurement extent. To identify domain boundaries for prediction (2), we followed Wheatley (2010) in defining domains of scale as regions where the relationship between predictor and response extents was statistically homogeneous. We adapted this concept to continuous extent using hierarchical GAMs (Pedersen et al. 2019, 2020) and identified domain boundaries where first derivatives indicated qualitative shifts in the relationship—specifically where the first derivative changed from significantly positive to non-significant, significantly negative to non-significant, or reversed direction (95% CI excluding zero). Derivatives were calculated using finite differences with a 1 ha step size and uncertainty propagated through the coefficient covariance matrix (n = 1000 simulations).

## Results

We detected 92 species at 392 sites, 79 of which were passerines (family Passeriformes; Table S1) and included in our measure of species richness, where mean site-level richness was 5.4 (SD = 2.2). We modelled species richness as a response of landscape heterogeneity, using either negative binomial or Poisson GLMM, at 13 response extents (estimated to be from 0.8 to 38 ha; 50–350 m radius). Landscape configuration metrics were set at their scale of effect for the final models (65 models). Among the final models, all metrics were best fit as linear and non-interacting terms; none included an interaction between the configuration metric and proportion uplands or the metric’s polynomial. Full model statistics are in Table 1. Species richness increased asymptotically with surveyed area, from a mean of 1 species (SD = 0.94) at 1 ha to 6 species (SD = 2.3) at 38 ha (Fig. S1). The plateau in species accumulation reflects both ecological processes (territory density, proximity of diverse habitat) and detection constraints inherent to passive acoustic monitoring. Beyond approximately 20 ha, the curve flattens as survey extents begin to exceed the effective detection radius for most species, particularly those with quieter songs or in closed-canopy habitats (Yip et al. 2017).

**Table 1:** Model fit statistics for each configuration metric at its scale of effect (landscape extent), depending on the spatial extent at which the response, species richness, is measured

Configuration metric	Response extent (ha)	Landscape extent (ha)	Parameters	Estimate	Std. Error	Pr( >|z|)
CAI CV	0.79	75	full model	-0.211	0.121	0.082
	1.8	65	full model	-0.011	0.080	0.891
	3.1	35	full model	0.074	0.062	0.238
	4.9	40	no survey year	0.066	0.050	0.186
	7.1	70	no survey year	-0.047	0.044	0.277
	10	39	no survey year	0.016	0.030	0.587
	13	37	no survey year	0.070	0.030	0.021
	16	37	full model	0.055	0.028	0.048
	20	36	full model	0.041	0.027	0.122
	24	35	full model	0.038	0.026	0.154
	28	34	no autocov	0.048	0.026	0.064
	33	33	no survey year or autocov	0.044	0.026	0.085
	38	39	no survey year or autocov	0.034	0.026	0.193
CONTAG	0.79	75	no survey year	-0.148	0.138	0.284
	1.8	73	no survey year	-0.095	0.106	0.370
	3.1	64	no survey year	0.001	0.084	0.991
	4.9	71	no survey year	-0.036	0.069	0.601
	7.1	40	no survey year or autocov	-0.047	0.056	0.396
	10	39	no survey year	-0.082	0.043	0.055
	13	44	no survey year	-0.089	0.040	0.026
	16	43	no survey year or autocov	-0.029	0.037	0.433
	20	42	Poisson, no autocov	-0.035	0.035	0.316
	24	48	full model	-0.043	0.034	0.207
	28	40	no autocov	-0.024	0.033	0.467
	33	46	no autocov	-0.032	0.034	0.340
	38	45	no autocov	-0.032	0.034	0.343
Edge density	0.79	0.79	no survey year	0.118	0.060	0.050
	1.8	1.8	full model	0.127	0.051	0.012
	3.1	3.2	no survey year	0.080	0.048	0.097
	4.9	11	no survey year	0.080	0.045	0.079
	7.1	14	no survey year	0.098	0.039	0.013
	10	9.7	full model	0.092	0.031	0.003
	13	13	full model	0.075	0.030	0.013
	16	16	full model	0.045	0.030	0.132
	20	36	full model	0.039	0.032	0.227
	24	35	full model	0.041	0.031	0.196
	28	34	no survey year or autocov	0.044	0.031	0.152
	33	33	no survey year or autocov	0.048	0.031	0.119
	38	39	no survey year or autocov	0.050	0.031	0.105
PAFRAC	0.79	75	no autocov	-0.175	0.110	0.111
	1.8	73	no autocov	-0.070	0.083	0.398
	3.1	72	no autocov	-0.086	0.070	0.216
	4.9	63	no autocov	-0.103	0.054	0.055
	7.1	62	no autocov	-0.073	0.045	0.104
	10	13	full model	0.065	0.035	0.065
	13	13	no autocov	0.055	0.032	0.086
	16	16	full model	0.082	0.030	0.006
	20	36	Poisson, no autocov	0.045	0.024	0.063
	24	35	full model	0.043	0.024	0.077
	28	34	full model	0.050	0.023	0.030
	33	33	no autocov	0.048	0.023	0.037
	38	39	no autocov	0.053	0.024	0.030
SHDI	0.79	1.4	full model	0.691	0.252	0.006
	1.8	1.8	full model	0.494	0.193	0.010
	3.1	3.2	full model	0.246	0.154	0.111
	4.9	15	full model	0.194	0.108	0.073
	7.1	18	full model	0.187	0.092	0.041
	10	19	full model	0.178	0.073	0.014
	13	79	full model	0.154	0.066	0.019
	16	78	no survey year	0.100	0.059	0.092
	20	79	no survey year	0.123	0.057	0.030
	24	35	no survey year	0.107	0.054	0.050
	28	31	no survey year	0.096	0.054	0.075
	33	33	full model	0.093	0.053	0.081
	38	79	full model	0.094	0.054	0.084

All models are fit with a negative binomial distribution unless otherwise specified. Full model indicates all covariates were used, including the spatial autocovariate and survey year random effect. Estimate, std. error, and p-values refer to the marginal effect of the configuration metric. CAI CV, core area index coefficient of variation; CONTAG, contagion; PAFRAC, perimeter-area fractal dimension; SHDI, Shannon diversity

### Identifying scale of effects

Scale of effects were clearer for some combinations of response and landscape extents. For instance, edge density was consistently positively correlated with species richness, but the scale of effect was stronger when the bird community was surveyed within 1.8 ha—75 m radius—(landscape extent: 1.8 ha, *ꞵ* = 0.164, 95% CI: 0.044, 0.284) than within 28 ha—300 m radius—(landscape extent: 34 ha, *ꞵ* = 0.044, 95% CI: -0.016, 0.105) (Fig. S3). For several metrics, like edge density and Shannon diversity, broad ranges of landscape extents were statistically significantly associated with species richness.

### Edge density

**Fig. 2:**
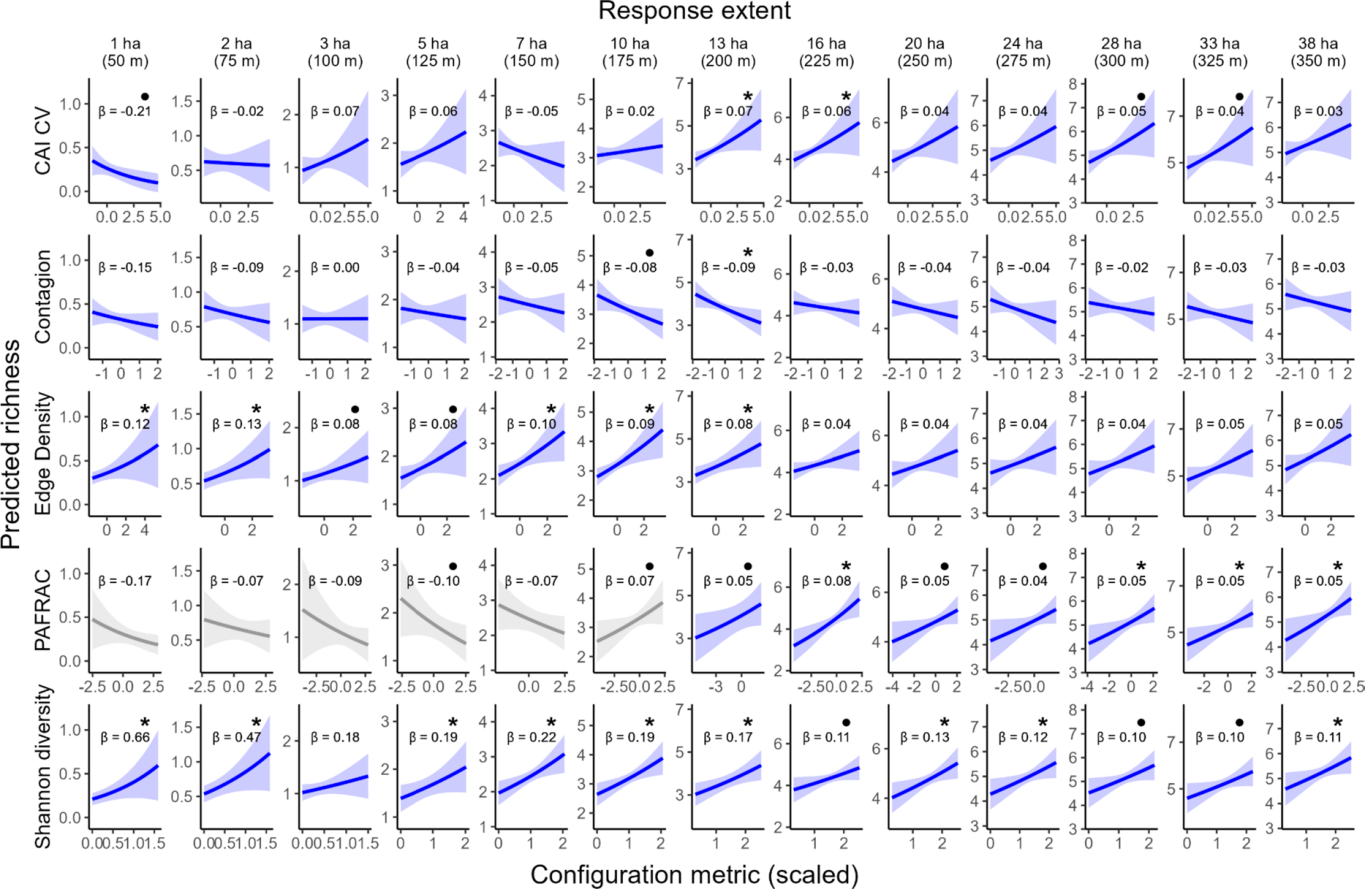
Predicted species richness responds differently to each configuration metric, depending on the extent in which birds are counted. Y-axes are constant for each response extent (column-wise); note the increase in species richness as response increases, where within 1 ha richness ranges from 0 to 1.2 species, and from 3 to 8 within 38 ha. Configuration metrics are at their unique scale of effect extent for each response extent. Solid lines show mean predictions, shaded areas are 95% confidence intervals, beta values (ꞵ) are coefficient estimates for the metrics, and stars (*) indicate statistical significance at alpha < 0.05 and bullets (•) at 0.05 < alpha < 0.1. Grey slopes for PAFRAC indicate extents where more than 40% of sites did not have the minimum 10 patches to compute the metric. All other parameters are held at their mean

**Fig. 3:**
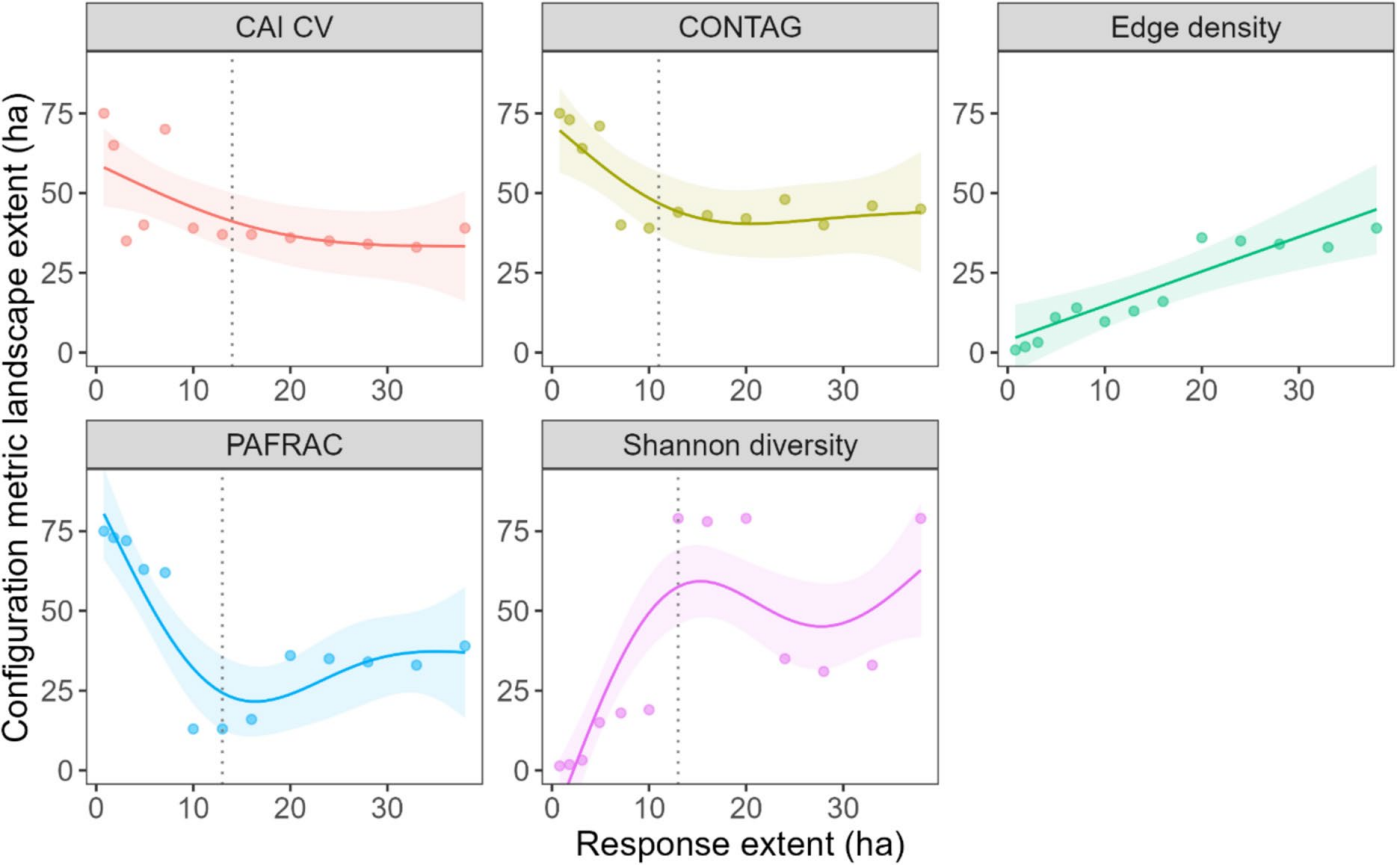
Relationship between landscape extents (configuration metric scale of effect) and response extents (species richness measurement extent) from hierarchical generalized additive models. Each panel shows how the spatial extent at which a configuration metric best predicts species richness changes with the spatial extent at which species richness is measured. Points represent the optimal landscape extent identified from scale of effect analyses for each response extent. Shaded regions indicate 95% confidence intervals. A 1:1 relationship (diagonal line from origin) would indicate that landscape and response extents match at all extents (spatial autocorrelation dominates), as observed for edge density. Departures from 1:1 and changes in slope indicate scale-dependent relationships and domain boundaries. CAI CV: core area index coefficient of variation; CONTAG: contagion; PAFRAC: perimeter-area fractal dimension. Vertical dotted lines represent domain transitions

Edge density as a predictor for species richness was consistently positive, but effects were not statistically significant at broader response extents (16–40 ha; 225–350 m radius), where 95% confidence intervals overlapped 0 (Table 1; Fig. 2). Scale of effects were consistently around the same extents as those in which bids were counted (Fig. 3).

### Core area index coefficient of variation

There was a single scale domain for CAI CV, from the response extent of 14 ha to greater, but the predictor was only statistically significant for two of the seven models within the scale domain. Across these extents, CAI CV was a positive driver of species richness (Fig. 2), and the scale of effects were around 36 ha, indicating consistent scale of effect relationships across broader response extents (Fig. 3).

### Perimeter-area fractal dimension

Perimeter-area fractal dimension (PAFRAC) had a negligeable effect on species richness at fine response extents (below 8 ha; Table 1). Within the 10–38 ha response extents (175–350 m radius), PAFRAC was consistently positive, but the predictor’s 95% CI in four of the eight models at those extents overlapped 0. For these eight models where PAFRAC was positively related with species richness, the predictor’s scale of effects were between 13 and 39 ha.

### Shannon diversity index

Shannon diversity was consistently positively associated with bird species richness across all response extents (Fig. 2). Largest effect sizes occurred at the two finest extents (1 and 2 ha; 50 and 75 m radius; Fig. S4). There were two scale domains for this metric, transitioning at the response extent of 13 ha. Within the first domain, the 95% confidence intervals for five out of six extents at which the response was measured did not overlap 0, and scale of effects occurred between 1 and 20 ha landscape extents. In the second domain, the scale of effects were between 31 and 79 ha, where the average scale of effect extent was 59 ha (Fig. 3).

### Contagion

CONTAG was consistently negatively associated with bird species richness at response extents greater than 7 ha (150 m radius), but the predictor’s 95% confidence intervals overlapped 0 at all extents except at the 13 ha (200 m radius) response extent (landscape extent: 44 ha; ꞵ = -0.09, 95% CI: -0.167, -0.011). The signal was clearest and most consistent for response extents 8–38 ha (150–350 m), where CONTAG scale of effects were within 39–48 ha, corresponding to the second response scale domain (Table 2).

**Table 2:** Domain boundaries identified from first derivative transitions in hierarchical generalized additive models (HGAM) relating landscape extents to response extents for five landscape configuration metrics

Configuration metric	Response extent (ha)	1st derivative lower 95% CI	1st derivative upper 95% CI	Domain change	Selected
CAI CV	14	–2.1	0.0	True	Yes
CONTAG	11	–2.6	0.0	True	Yes
Edge density	None	none	none	none	none
PAFRAC	13	0.2	0.9	True	Yes
SHDI	13	–0.06	2.5	True	Yes
SHDI	22	–3.5	–0.01	True	No
SHDI	24	–2.5	0.0	True	No

Domain boundaries were identified where the first derivative transitioned between significantly positive (95% CI excluding zero above), non-significant (95% CI overlapping zero), or significantly negative (95% CI excluding zero below), but only when the transitioning slope 95% CI did not overlap 0. Response extent indicates the spatial extent at which species richness was measured where the domain boundary occurred. The derivative state describes the relationship before and after each boundary

### Predictors controlling for productivity

Proportion uplands varied between 0–1 with a long left tail; the mean was 0.947, median = 0.997, SD = 0.106 (Fig. S2). It was mostly statistically non-significant across models, especially at broad response extents. Growing degree days (GDD) was always positive and statistically significant, with strongest effect sizes at fine response extents (Fig. S4).

### Scale dependence more important than spatial autocorrelation

From the scale of effect consistency model, where each predictor's scale of effect extents are mapped against response extents, four of the five metrics showed scale dependence on both the response (species richness) and predictor variables (configuration metrics; Fig. 3). Edge density showed a consistently positive derivative across all response extents, with confidence intervals excluding zero throughout, indicating a 1:1 relationship with no domain structure (prediction 1). CAI CV, CONTAG, PAFRAC, and Shannon diversity showed transitions in derivative sign or significance (Fig. S5), indicating domain boundaries where relationships shifted qualitatively (prediction 2). Domain boundaries occurred at 14 ha response extent for CAI CV, 11 ha for CONTAG, 13 ha for PAFRAC and Shannon diversity (Table 2).

Applying our derivative-based criteria, Shannon diversity index showed multiple transitions of the first derivative around zero (Table 2, Fig. S5). However, only the transition at 13 ha represented a substantive shift from a strong positive relationship (0–13 ha) to oscillations near zero (13–40 ha). The 95% CI of transitions after 13 ha nearly all overlapped 0, so we grouped these fluctuations into a single domain for parsimony.

Four of the five landscape configuration metrics exhibited two scale domains: domain 1 at response extents < 10–14 ha, and domain 2 at response extents > 10–14 ha. Edge density showed no domain structure, with landscape extents matching response extents across the entire gradient (0–40 ha). Area-sensitive metrics (CAI CV, CONTAG, and PAFRAC) had landscape scale of effect extents substantially greater than response extents in domain 1, with this mismatch decreasing in domain 2 (Fig. 3). Edge density showed equivalent landscape and response extents throughout.

**Fig. 4:**
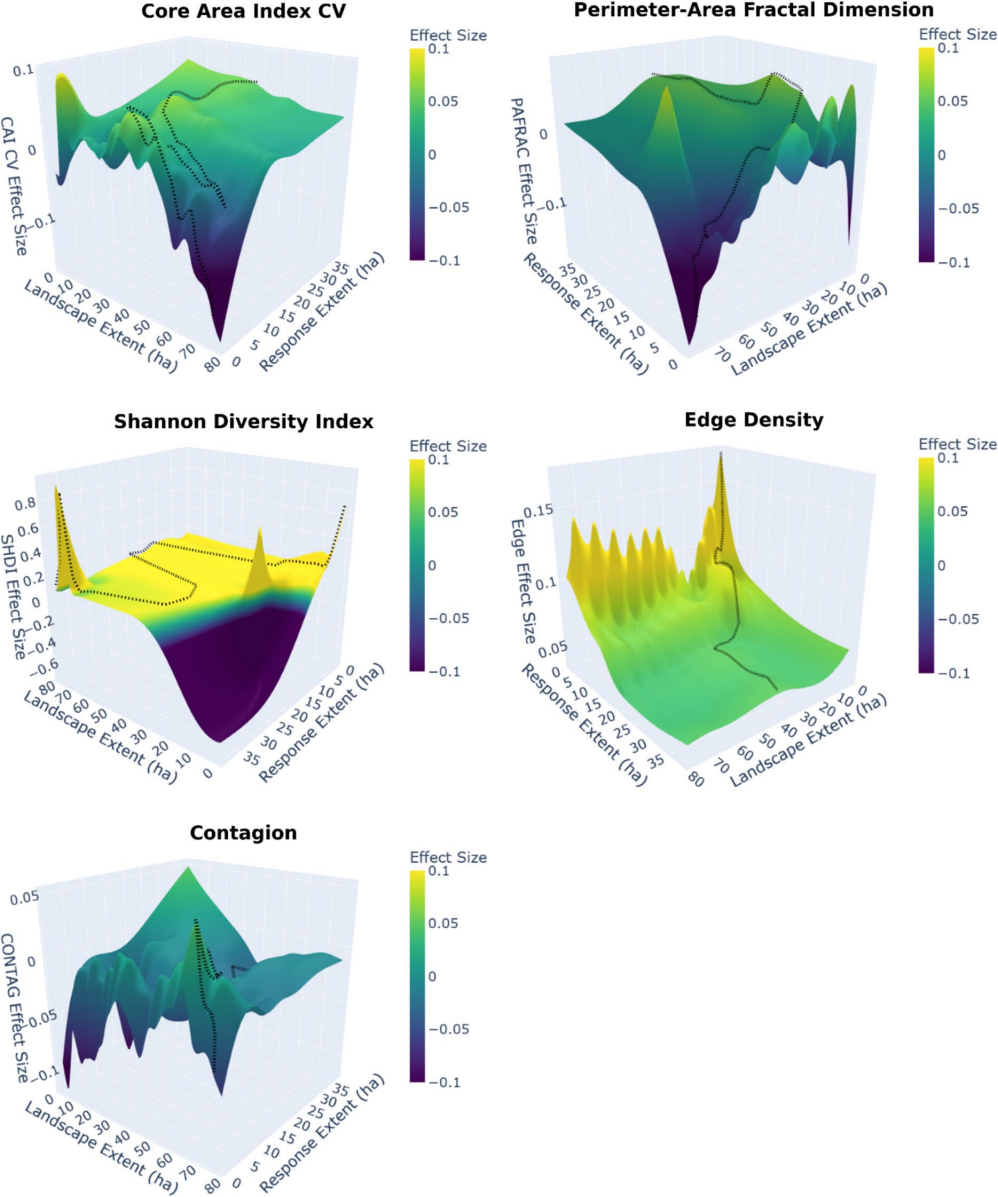
Effect sizes for landscape configuration metrics are unstable at fine spatial extents but converge at broader extents, demonstrating that extent selection determines ecological inference. Surfaces show effect sizes (scaled and centred coefficients) across all combinations of response extent (species richness measurement area) and landscape extent (metric calculation area). Dotted lines trace the scale of effect for each response extent. Note that axes are oriented to maximise surface visibility and vary between panels. For PAFRAC, effect sizes at fine extents reflect a reduced subset of sites where the metric was calculable (see Fig. S6). CAI CV: core area index coefficient of variation; CONTAG: contagion; PAFRAC: perimeter-area fractal dimension; SHDI: Shannon diversity index

The scale of effect models mapped to distinct ranges of landscape metric values across domains. Edge density increased rapidly from x̅ = 71 m/ha (SD = 140 m/ha) at the finest extents to x̅ = 154 m/ha (SD = 75 m/ha) at ~ 20 ha, then stabilized at x̅ = 155 m/ha (SD = 70 m/ha). CAI coefficient of variation in domain 1 ranged from x̅ = 95% (SD = 59%) to x̅ = 111% (SD = 62%), while in domain 2 averaged 122% (SD = 57%). PAFRAC values in domain 1 averaged x̅ = 1.22 (SD = 0.048), with similar values in domain 2 ranging from x̅ = 1.17 (SD = 0.057) to x̅ = 1.20 (SD = 0.058). Shannon diversity was low in domain 1 (x̅ = 0.43, SD = 0.44) and substantially higher in domain 2 (x̅ = 1.1, SD = 0.5). CONTAG showed similar values between domains: domain 1 landscape extents (60–80 ha) averaged 64% (SD = 7%), while domain 2 landscape extents (39–45 ha) averaged 65% (SD = 8%). The joint dependence of effect sizes on both response and landscape extents is visualized in Fig. 4, where 3D surface plots reveal that effect sizes for area-sensitive metrics (CAI CV, PAFRAC, CONTAG) oscillate erratically at fine response extents (< 5 ha) before stabilizing across the domain boundary, while edge density and Shannon diversity show more consistent surfaces. PAFRAC was incalculable for all of the 392 sites at the finest landscape extent (50 m radius), but calculable for 60% of sites at 200 m radius and all the sites at the broadest landscape extent (Fig. S6), meaning models at fine extents reflect a non-random subset of the most heterogeneous sites.

## Discussion

We developed a framework to empirically parse spatial dependence of survey area (response extent for species richness) and measurement of configuration metrics (landscape extent) when relating spatial heterogeneity to species richness in managed forests. Landscape scale of effects—extents where metrics had strongest associations with species richness—were unique for each metric and varied systematically with response extent (Gustafson 1998). Four of five landscape configuration metrics converged on domain boundaries at 11–14 ha (175–210 m radius) despite distinct scaling behaviors and measurement properties. This convergence across independent metrics reveals a fundamental ecological transition rather than metric-specific artifacts. Below this threshold, within-stand heterogeneity—indexed by edge density and compositional diversity—drives species richness through localized resource diversification. Above it, mosaic configuration—indexed by area-sensitive metrics—becomes influential, reflecting processes operating across the local neighborhood rather than within individual harvest plots. The boundary aligns with the average spatial extent (~ 186 m radius; 11 ha) separating harvest interiors from surrounding older forests, suggesting this threshold marks where species assembly shifts from within-patch resource partitioning to landscape mosaic complementarity. Across all metrics, none of the 65 models included significant interactions between configuration metrics and proportion upland, likely reflecting the limited variation in upland proportion across sites (80–100%). In this range, configuration effects on species richness operate independently of local upland proportion, consistent with expectations when cover of the most productive forest type is well above the percolation threshold and uplands remain broadly connected (Villard and Metzger 2014).

### First domain: within-stand heterogeneity

Below 11–14 ha, edge density and Shannon diversity were the strongest and most consistent predictors of species richness, with both metrics' scale of effects within 20 ha landscape extents. Within-block configurational and compositional heterogeneity, driven by irregular harvest boundaries, variable retention, riparian buffers, and vegetated linear features from energy sector activity (Zenner 2000; Braithwaite and Mallik 2012; Harper et al. 2015; Viliani and Nielsen 2025), increase species richness of post-disturbance bird communities (MacArthur 1965). These features diversify foliage height, age class, and tree species composition in otherwise homogenous stands. Variable retention supports early-seral species (Schieck et al. 2000; Lebeuf-Taylor et al. 2025), and riparian buffers increase species richness in regenerating areas (Triquet et al. 1990), and, when sufficiently large, retain forest-associated species like the Ovenbird (*Seiurus aurocapilla*; Hannon 2000). Vegetated linear features attract open-habitat species, enhancing local diversity as harvest canopies close (Kalukapuge et al. 2024).

Edge density's strongest effects at fine extents (x̅ = 71 m/ha, SD = 140 m/ha) demonstrate that even modest edge increases are important for species richness in regenerating stands. In naturally patchy landscapes, boreal songbirds increase in abundance and species richness with edge density (Parker et al. 2005; Ball et al. 2009), where negative effects from brood parasites and predators common in agricultural systems do not dampen diversity gains (Bayne and Hobson 1997; Ball et al. 2009). Between contrasting tree age and height patches, downed woody debris and reduction in canopy density (Burton 2002) increases access to complementary resources, such as foraging substrates, nesting sites, and microclimates, within concentrated areas (Dunning et al. 1992; Van Wilgenburg et al. 2001; Ries et al. 2004). High edge density can be detrimental to interior forest specialists (Schieck and Song 2006), but these species were largely absent from our study, where ARUs were placed in regenerating harvests that primarily supported disturbance-adapted and edge-tolerant species. Edge density exhibited 1:1 scaling between landscape and response extents, indicating edge effects operate at the immediate species response extents regardless of the extent at which edge density is measured. Shannon diversity showed contrasting behavior: below 4 ha response extent, scale of effects matched response extents, but beyond this threshold, optimal landscape extents stabilized at 60–75 ha. This divergence suggests edge effects reflect immediate local processes, while compositional diversity integrates vegetation heterogeneity across broader spatial extents to influence local species richness.

### Second domain: mosaic-level configuration

Beyond 13 ha (200–350 m radius), configurational metrics characterizing mosaic properties—shape complexity (PAFRAC), patch size variability (CAI CV), and contagion (CONTAG)—emerged as stronger predictors of richness (Mladenoff et al. 1993; Cumming and Vernier 2002; Mori et al. 2017). These metrics capture structural properties of the landscape mosaic in which harvest plots are embedded, rather than within-stand heterogeneity. The consistent domain boundary at 11–14 ha across these metrics, where statistical support strengthened abruptly (except for contagion), suggests a shift from within-stand to mosaic-level structural properties influencing species richness.

PAFRAC showed scale-dependent relationships with species richness, with weakly supported negative relationships at response extents below 9 ha, shifting to consistent positive effects above 13 ha. High PAFRAC values reflect mosaics with complex patch boundaries and high edge-to-area ratios. Unmanaged North American forests have higher fractal dimension than forests logged prior to the 1990s (Mladenoff et al. 1993; Vernier 1995), when conventional forestry created geometrically simple harvest blocks. Alberta's current harvest mosaic includes legacy patterns from that era alongside more recent natural disturbance emulation practices, providing a gradient in shape complexity. This variation enables comparison of mosaic-level configuration effects on species richness—our finding that PAFRAC positively predicts richness at broad response extents aligns with previous observations that shape complexity correlates with higher songbird abundance (Brennan and Schnell 2005). The domain 1 pattern is too statistically weak for confident interpretation; resolving whether shape complexity operates differently within stands versus in broader mosaics of harvest and unharvested patches requires targeted replication.

Variation in mean patch size (CAI CV) was non-significant at response extents < 10 ha but consistently positive and statistically significant for two extents beyond this threshold. Where detectable, scale of effects for CAI CV occurred at 35–40 ha landscape extents—substantially broader than response measurement extents. The 12 ha detection threshold likely reflects two processes: (1) minimum area requirements for reliable metric calculation (McGarigal et al. 2023), and (2) the spatial extent across which variation in patch core area influences species distributions. In our study region, low CAI CV characterizes harvest mosaics with uniform patch sizes (e.g., repeated standard cutblock geometries), while high CAI CV reflects varied patch sizes from mixing old-growth remnants, variable retention harvests, and different harvest practices. Uniform harvest plot geometries support lower diversity than varied patch sizes.

Contagion (CONTAG)—indexing patch aggregation and contiguity—showed consistent negative trends but weak statistical support (95% CI overlapping zero at all response extents except 13 ha). In a forestry context, high contagion does not support higher species richness (Mori et al. 2017). As an inverse measure of configurational heterogeneity, low contagion values characterize dispersed, intermixed patch configurations. Alberta's harvest mosaic has shifted toward larger contiguous harvest units: 95th percentile cutblock size increased from 30 ha (1960s) to 66 ha (2010) despite stable mean harvest size (ABMI 2024). This trend toward large aggregated harvests (high CONTAG) aligns with goals of spatially condensing disturbances to maintain large undisturbed forest tracts for sensitive species (Tittler et al. 2001; Atwell et al. 2008), but our findings suggest potential costs for overall songbird community richness in regenerating stands. Low contagion—achieved through spatially dispersed harvest units—may benefit local diversity but requires disturbing larger total areas under fixed annual timber yields. This creates a fundamental conservation tension: dispersed harvests enhance diversity in regenerating stands while potentially fragmenting mature forest required by area-sensitive species like the regionally declining Black-throated Green Warbler (*Setophaga virens*; Hart et al. 2025). With increasing annual harvest volumes (ABMI 2024), understanding scale-dependent contagion effects across different species groups is critical for reconciling competing objectives.

### Species richness responds to broader extents of landscape configuration

Scale of effects occurred at broader landscape extents than response measurement extents for most metrics. When richness was measured within 1–8 ha, PAFRAC's scale of effect occurred at 60–75 ha; similar mismatches characterized CAI CV and Shannon diversity. This parallels single-species studies where habitat use integrates information across broader extents than selection occurs (Malt and Lank 2009; Adams et al. 2024). This mismatch between response and landscape extents has critical implications for interpreting effect sizes of predictors across extents, stemming from the modifiable areal unit problem (MAUP; Openshaw 1983): landscape metrics' statistical properties change systematically with measurement extent. Edge density exemplifies this constraint—mean values increased from 71 m/ha (SD = 140 m/ha) at fine extents to 154 m/ha (SD = 75 m/ha) at intermediate extents before plateauing, while variance decreased nearly twofold. Declining effect sizes across response extents (edge density β = 0.13 at 2 ha vs. β = 0.04 at 28 ha) reflect both genuine ecological scaling and MAUP-driven compression of predictor variance. Direct cross-scale comparison of effect magnitudes requires examining the distribution of predictor values at each extent alongside the coefficients themselves. Shannon diversity was unique in maintaining a relatively stable coefficient of variation across measurement extents, enabling more straightforward cross-scale interpretation.

### Scale selection determines inference

Landscape configuration effects on species richness depend on both response and landscape measurement extents, with landscape configuration metrics operating at distinct spatial extents. Arbitrary response extent selection risks measuring diversity where ecological processes operate weakly, potentially missing threshold responses where relationships reverse across domains—as we observed with PAFRAC's sign shift between scale domains (With 2016; Zhang et al. 2024).

The 3D surface plots show the severity of this problem: effect sizes oscillate erratically at fine response extents (< 5 ha) for area-sensitive metrics (CAI CV, PAFRAC, CONTAG). These fluctuations do not represent genuine ecological reversals but rather zones of statistical instability where metrics are unreliable. PAFRAC, for example, requires a minimum of 10 patches (Hesselbarth et al. 2019), a threshold unmet by most sites at fine landscape extents. Models at these extents are therefore fit on a non-random subset of the most heterogeneous sites, and high model fit masks selection bias rather than reflecting genuine ecological signal. Some of this instability occurred at typical point count extents (7 ha; 150 m radius), highlighting that standard protocols may systematically sample less informative response extents. Indeed, the 50 m limited distance counts showed the strongest statistical instability; when the effect sizes and error bars are seen in context of the models from broader response extents, deriving ecological sense solely from models at finest extents is misleading.

At broader response extents, an analogous limitation arises from imperfect detection. Our measure of species richness is naive — it does not account for detection probability declining with distance from the ARU, particularly for quiet or closed-canopy associated species (Yip et al. 2017). Beyond ~ 20 ha, survey extents exceed the effective detection radius for most species, and the flattening species accumulation curve (Fig. S1) reflects detection constraints as much as ecological saturation. Our results at broad extents therefore represent a conservative snapshot of species present rather than true alpha diversity. More precise inference at these extents would require multi-ARU arrays deployed systematically within and around harvest blocks to maintain detection probability across the full survey area, should complete local richness be the objective.

### Implications for bioacoustic monitoring in managed forests

While avian-disturbance studies carefully select landscape extents, we demonstrate response extent selection matters equally. Traditional point count protocols—using fixed detection radii (50, 100, 150 m) or unlimited distances—implicitly assume either that single extents suffice or broader extents are universally optimal. Recent incorporation of continuous distance estimation (Dawson and Efford 2009; Yip et al. 2017; Edwards et al. 2023; Lebeuf-Taylor et al. 2025) enables systematic response extent variation. Directly measuring the role of spatial extent in response variables allows us to test ubiquitous spatial assumptions in bioacoustic protocols, and develop empirical methods to refine our understanding of how bird diversity occurs in space.

When measuring impacts of harvest plans on bird species richness, choice of survey extent determines which landscape features appear influential. Below 11–14 ha, within-stand heterogeneity matters: edge density and vegetation diversity drive species richness. Above this threshold, mosaic configuration matters: patch size variation and shape complexity predict richness. However, this depends on ARU placement and distance to the edge of harvest plots. With our study design, this suggests the threshold marks where survey extents begin capturing species at harvest-forest interfaces rather than only within regenerating stands. When measuring the impact of broader harvest plans on bird species richness, metrics like variation in mean patch size and shape complexity are more relevant, but only if birds are counted within broad survey areas (> 178–210 m radius). To target these metrics, aggregating species counts from multiple ARUs around harvest plots would address decreasing effective detection radius at these distances. If measuring effects of configurational heterogeneity on birds within cutblocks, detection radius should be limited to within the zone of interest. Selecting the relevant detection radius and landscape metric has direct implications for forest management planning and monitoring design.

## Conclusion

Our framework addresses a fundamental challenge in landscape ecology: how to identify ecologically relevant extents without a priori mechanistic predictions. Previous approaches to extent selection rely on species-specific traits (home range, dispersal distance) to predict relevant extents (Jackson and Fahrig 2015). Community-level processes lack such trait-based anchors, in part because all vegetation types in managed boreal forests are occupied by songbird species, precluding a meaningful definition of habitat amount for taxonomic richness; where monitoring targets a specific guild or vegetation type, an amount-based framework may be more appropriate. Our empirical approach—systematically varying both response and landscape extents to identify domains of scale (Wheatley 2010)—demonstrates that scale-dependent ecological transitions can be detected through convergence across independent metrics. The consistent 11–14 ha boundary across metrics with fundamentally different mathematical properties (edge density: perimeter-based; Shannon diversity: compositional; CAI CV: area distribution; PAFRAC: shape complexity) strengthens inference that this threshold reflects genuine ecological process shifts rather than statistical artifacts or MAUP effects. This methodological advance has direct conservation implications. Mosaic patterns generated by forestry demonstrably affect bird species richness in regenerating boreal harvests, but detecting and correctly interpreting these effects requires measuring community responses and landscape patterns at ecologically relevant extents. Arbitrary extent selection risks generating misleading inferences that propagate into ineffective management recommendations. Our single-ARU, multi-scale method offers a cost-effective approach to addressing scale-dependence, enabling forest managers to target the spatial extents where interventions will most influence songbird communities.

## Supplementary Information

Below is the link to the electronic supplementary material.

Supplementary file1 (DOCX 1014 KB)

Supplementary file2 (JPEG 526 KB)

Supplementary file3 (JPEG 1292 KB)

Supplementary file4 (JPG 154 KB)

Supplementary file5 (JPEG 3000 KB)

Supplementary file6 (JPEG 555 KB)

Supplementary file7 (JPEG 194 KB)

Supplementary file8 (DOCX 24 KB)

## Data Availability

Code to generate analyses and figures is available at https://github.com/IsabelleLebTay/boreal-songbird-scale-heterogeneity. Analyses were performed in R (R Core Team 2024) and Python 3.12, and can be reproduced using the post-processed data archived at 10.5683/SP3/RZ5DUQ. Raw avian data are publicly available on the WildTrax platform (https://www.wildtrax.ca). Raw vegetation and human footprint data are proprietary and owned by the Alberta Biodiversity Monitoring Institute (https://abmi.ca).
