# Supplementary material for: Configurational heterogeneity drives songbird diversity at distinct spatial scales in managed boreal forests
Source: Landsc Ecol. 2026 Mar 27;41(5):86. doi: 10.1007/s10980-026-02341-y (PMC13156193; doi:10.1007/s10980-026-02341-y)
Supplement: Supplementary file 1 — Supplementary file1 (DOCX 1014 KB) [file 10980_2026_2341_MOESM1_ESM.docx]

# Supplementary Material

Table S1. Detected species (92), their order, and number of sites in which they were detected, from a total of 392 sites. Only Passeriformes were included in the analysis.

| Common name | Scientific name | Order | Count |
| --- | --- | --- | --- |
| Alder Flycatcher | *Empidonax Alnorum* | Passeriformes | 146 |
| American Crow | *Corvus Brachyrhynchos* | Passeriformes | 7 |
| American Goldfinch | *Spinus Tristis* | Passeriformes | 3 |
| American Kestrel | *Falco Sparverius* | Falconiformes | 1 |
| American Redstart | *Setophaga Ruticilla* | Passeriformes | 12 |
| American Robin | *Turdus Migratorius* | Passeriformes | 136 |
| American Three-toed Woodpecker | *Picoides Dorsalis* | Piciformes | 2 |
| American Wigeon | *Mareca american* | Anseriformes | 1 |
| Baltimore Oriole | *Icterus Galbula* | Passeriformes | 1 |
| Bay-breasted Warbler | *Setophaga Castanea* | Passeriformes | 1 |
| Black and white Warbler | *Mniotilta Varia* | Passeriformes | 15 |
| Black-billed Magpie | *Pica Hudsonia* | Passeriformes | 1 |
| Black-capped Chickadee | *Poecile Atricapillus* | Passeriformes | 11 |
| Black-throated Green Warbler | *Setophaga Virens* | Passeriformes | 1 |
| Blue Jay | *Cyanocitta Cristata* | Passeriformes | 3 |
| Blue-headed Vireo | *Vireo Solitarius* | Passeriformes | 6 |
| Boreal Chickadee | *Poecile Hudsonicus* | Passeriformes | 3 |
| Brown Creeper | *Certhia Americana* | Passeriformes | 1 |
| Brown-headed Cowbird | *Molothrus Ater* | Passeriformes | 24 |
| Canada Goose | *Branta Canadensis* | Anseriformes | 4 |
| Canada Jay | *Perisoreus Canadensis* | Passeriformes | 63 |
| Canada Warbler | *Cardellina Canadensis* | Passeriformes | 1 |
| Cassin's Vireo | *Vireo Cassinii* | Passeriformes | 1 |
| Cedar Waxwing | *Bombycilla Cedrorum* | Passeriformes | 25 |
| Chipping Sparrow | *Spizella Passerina* | Passeriformes | 120 |
| Clay-colored Sparrow | *Spizella Pallida* | Passeriformes | 44 |
| Common Loon | *Gavia Immer* | Gaviiformes | 3 |
| Common Raven | *Corvus Corax* | Passeriformes | 17 |
| Common Redpoll | *Acanthis Flammea* | Passeriformes | 2 |
| Common Yellowthroat | *Geothlypis Trichas* | Passeriformes | 38 |
| Connecticut Warbler | *Oporornis Agilis* | Passeriformes | 2 |
| Dark-eyed Junco | *Junco Hyemalis* | Passeriformes | 98 |
| Downy Woodpecker | *Picoides pubescens* | Piciformes | 3 |
| Dusky Flycatcher | *Empidonax Oberholseri* | Passeriformes | 7 |
| European Starling | *Sturnus Vulgaris* | Passeriformes | 1 |
| Evening Grosbeak | *Coccothraustes Vespertinus* | Passeriformes | 1 |
| Fox Sparrow | *Passerella Iliaca* | Passeriformes | 2 |
| Golden-crowned Kinglet | *Regulus Satrapa* | Passeriformes | 7 |
| Greater Yellowlegs | *Tringa Melanoleuca* | Charadriiformes | 3 |
| Hairy Woodpecker | *Dryobates Villosus* | Piciformes | 4 |
| Hermit Thrush | *Catharus Guttatus* | Passeriformes | 72 |
| House Wren | *Troglodytes Aedon* | Passeriformes | 5 |
| Least Flycatcher | *Empidonax Minimus* | Passeriformes | 24 |
| LeConte's Sparrow | *Ammospiza Leconteii* | Passeriformes | 3 |
| Lesser Yellowlegs | *Tringa Flavipes* | Charadriiformes | 7 |
| Lincoln's Sparrow | *Melospiza Lincolnii* | Passeriformes | 133 |
| Magnolia Warbler | *Setophaga Magnolia* | Passeriformes | 35 |
| Mountain Chickadee | *Poecile Gambeli* | Passeriformes | 4 |
| Mourning Warbler | *Geothlypis Philadelphia* | Passeriformes | 36 |
| Nashville Warbler | *Leiothylpis Ruficapilla* | Passeriformes | 2 |
| Northern Flicker | *Colaptes Auratus* | Piciformes | 14 |
| Northern Waterthrush | *Parkesia Noveboracensis* | Passeriformes | 10 |
| Olive-sided Flycatcher | *Contopus Cooperi* | Passeriformes | 20 |
| Orange-crowned Warbler | *Leiothlypis Celata* | Passeriformes | 104 |
| Ovenbird | *Seiurus Aurocapilla* | Passeriformes | 37 |
| Pacific Wren | *Troglodytes Pacificus* | Passeriformes | 1 |
| Palm Warbler | *Setophaga Palmarum* | Passeriformes | 25 |
| Pileated Woodpecker | *Dryocopus Pileatus* | Piciformes | 5 |
| Pine Siskin | *Spinus Pinus* | Passeriformes | 62 |
| Purple Finch | *Haemorhous Purpureus* | Passeriformes | 2 |
| Red Crossbill | *Loxia Curvirostra* | Passeriformes | 2 |
| Red-breasted Nuthatch | *Sitta Canadensis* | Passeriformes | 27 |
| Red-eyed Vireo | *Vireo Olivaceus* | Passeriformes | 98 |
| Red-winged Blackbird | *Agelaius Phoeniceus* | Passeriformes | 2 |
| Rose-breasted Grosbeak | *Pheucticus Ludovicianus* | Passeriformes | 38 |
| Ruby-crowned Kinglet | *Regulus calendula* | Passeriformes | 47 |
| Ruffed Grouse | *Bonasa Umbellus* | Galliformes | 10 |
| Savannah Sparrow | *Passerculus Sandwichensis* | Passeriformes | 1 |
| Say's Phoebe | *Sayornis saya* | Passeriformes | 1 |
| Solitary Sandpiper | *Tringa Solitaria* | Charadriiformes | 2 |
| Song Sparrow | *Melospiza Melodia* | Passeriformes | 2 |
| Swainson's Thrush | *Catharus Ustulatus* | Passeriformes | 177 |
| Swamp Sparrow | *Melospiza Georgiana* | Passeriformes | 4 |
| Tennessee Warbler | *Leiothlypis Peregrina* | Passeriformes | 140 |
| Townsend's Solitaire | *Myadestes Townsendi* | Passeriformes | 9 |
| Varied Thrush | *Ixoreus Naevius* | Passeriformes | 40 |
| Vesper Sparrow | *Pooecetes Gramineus* | Passeriformes | 4 |
| Warbling Vireo | *Vireo Gilvus* | Passeriformes | 88 |
| Western Flycatcher | *Empidonax Difficilis* | Passeriformes | 1 |
| Western Tanager | *Piranga Ludoviciana* | Passeriformes | 18 |
| Western Wood Pewee | *Contopus Sordidulus* | Passeriformes | 11 |
| White-breasted Nuthatch | *Sitta Carolinensis* | Passeriformes | 2 |
| White-crowned Sparrow | *Zonotrichia Leucophrys* | Passeriformes | 29 |
| White-throated Sparrow | *Zonotrichia Albicollis* | Passeriformes | 274 |
| White-winged Crossbill | *Loxia Leucoptera* | Passeriformes | 20 |
| Wilson's Snipe | *Gallinago Delicata* | Charadriiformes | 65 |
| Wilson's Warbler | *Cardellina Pusilla* | Passeriformes | 12 |
| Winter Wren | *Troglodytes Hiemalis* | Passeriformes | 10 |
| Yellow Warbler | *Setophaga Petechia* | Passeriformes | 15 |
| Yellow-bellied Flycatcher | *Empidonax Flaviventris* | Passeriformes | 15 |
| Yellow-bellied Sapsucker | *Sphyrapicus Varius* | Piciformes | 3 |
| Yellow-rumped Warbler | *Setophaga Coronata* | Passeriformes | 129 |


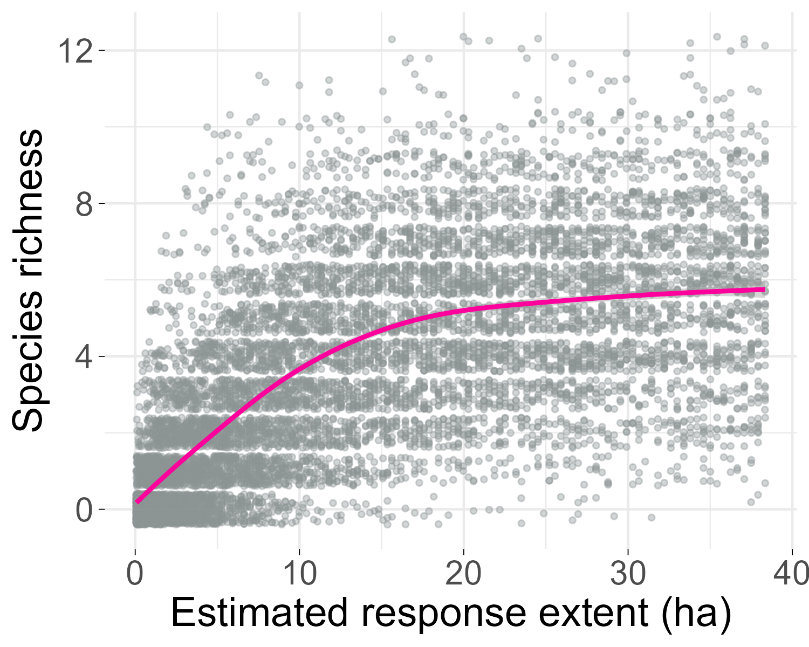


**Fig. S1** Species richness accumulation with response extent around acoustic point count locations (n = 392 sites). Fitted curve is a smooth estimated relationship between species richness and response extent. Response extents beyond 38.5 ha (350 m radius) exceed the maximum detection distance for many species, particularly those with quieter songs or in closed-canopy forest cover, though some species with louder territorial vocalizations in open areas remain detectable at these distances [(Yip et al. 2017)](https://www.zotero.org/google-docs/?broken=UkDqW0).


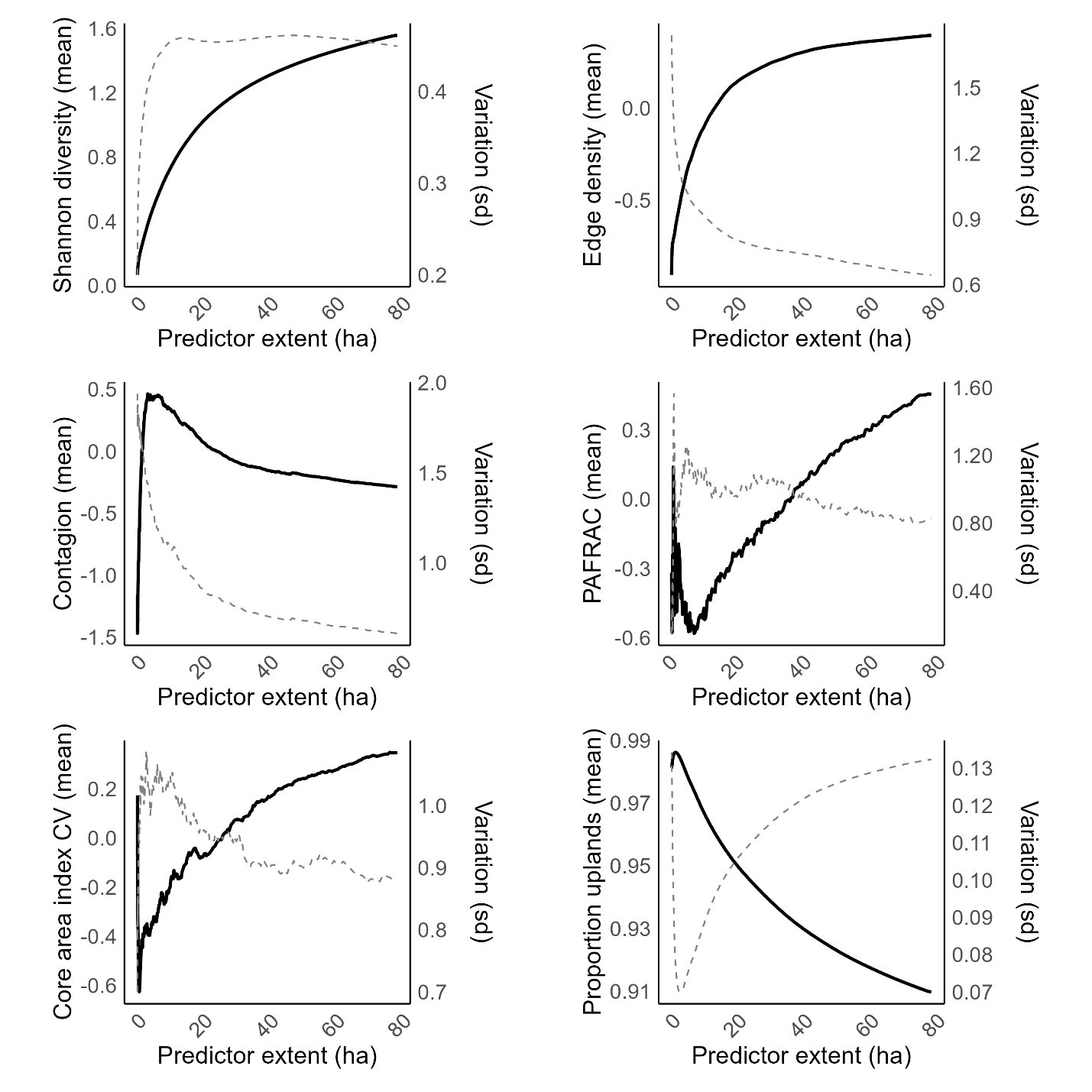


**Fig. S2** Each predictor varies in their mean (solid lines) and variance (dotted line) as the extent of measurement increases. Secondary y-axes are not on the same scale as primary y axes.


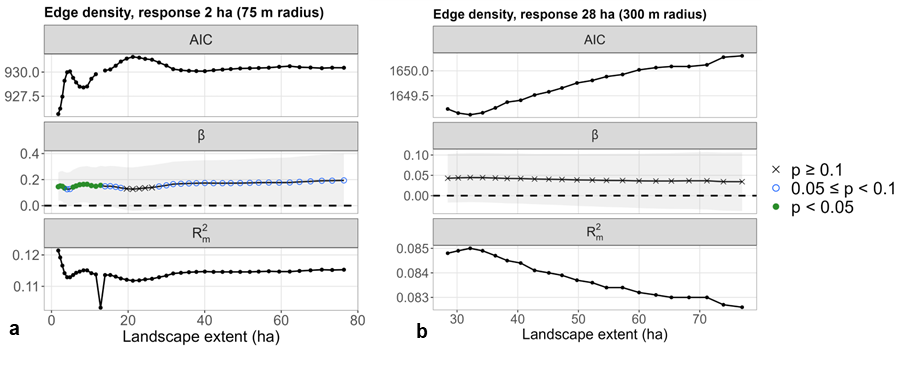


**Fig. S3** Scale-dependent effects of edge density on bird species richness vary with response extent**.** Model performance metrics (AIC, standardized coefficient β with 95% CI, and marginal R²) for edge density effects on species richness measured within (a) 1.77 ha (75 m radius) and (b) 28 ha (300 m radius), across landscape extents ranging from 0.8–80 ha. Point colors and shapes indicate significance levels of β. At the smaller response extent (a), edge density effects are significant at fine landscape extents (<5 ha) and become marginal at intermediate scales, while model fit (R²ₘ) peaks at the finest scale. At the larger response extent (b), edge density effects are consistently non-significant across all landscape extents, with minimal variation in model performance.

**
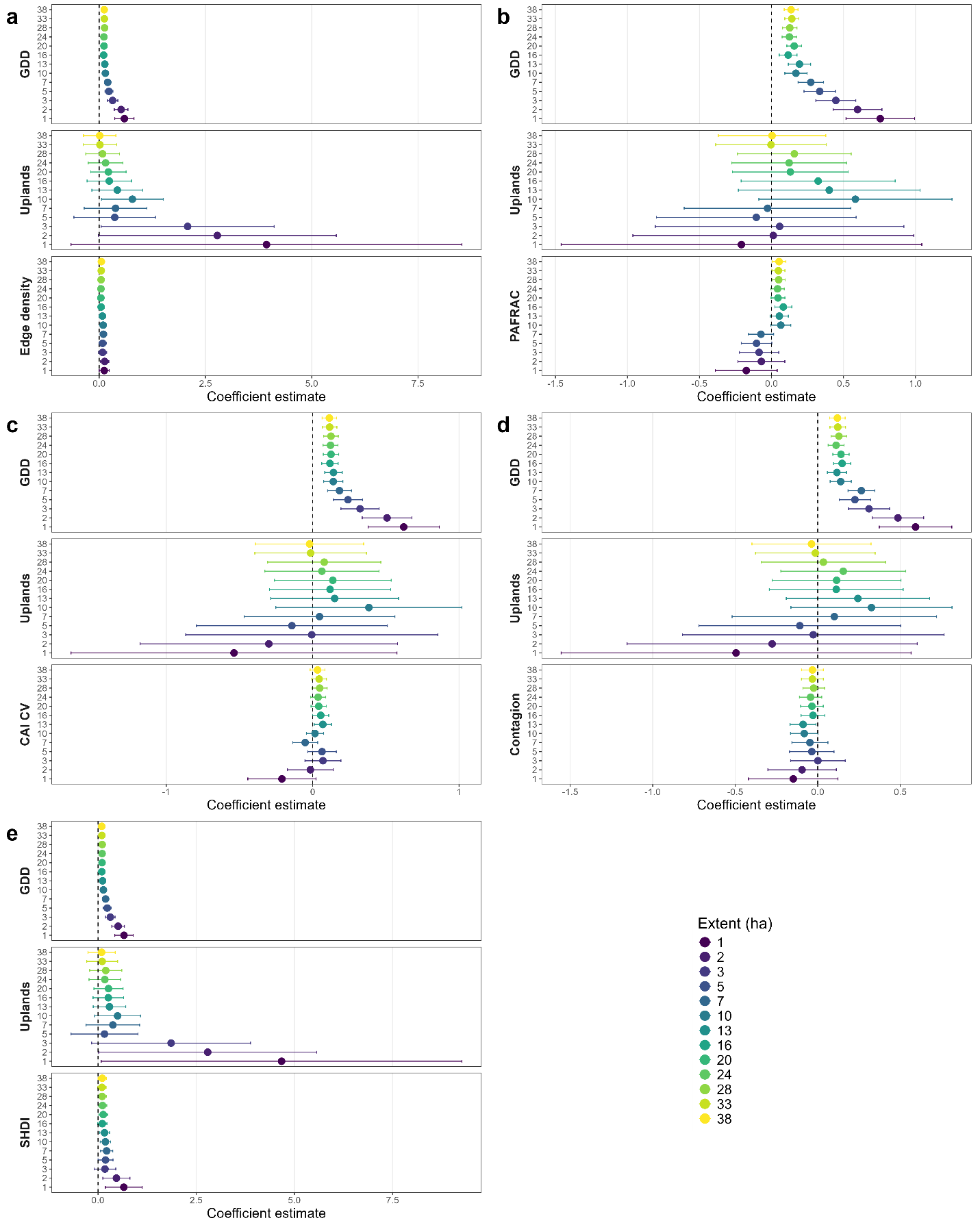
**

**Fig. S4** Coefficient estimates for all fixed effects across response extents, with each landscape configuration metric measured at its scale of effect. Each panel shows estimates for growing degree days (GDD), proportion upland, and the focal configuration metric: (a) edge density, (b) PAFRAC, (c) CAI CV, (d) contagion, (e) Shannon diversity index. Points represent coefficient estimates (log-link scale) with 95% confidence intervals from separate generalized linear mixed models (Poisson or negative binomial distribution) fit at each response extent (colour gradient, in ha). Configuration metrics were held at their identified scale of effect extent for each response extent (see Methods). Note that x-axis scales differ across panels as metrics were not standardized and were evaluated in separate models. The dashed vertical line indicates zero. CAI CV: core area index coefficient of variation; CONTAG: contagion; PAFRAC: perimeter-area fractal dimension; SHDI: Shannon diversity index.


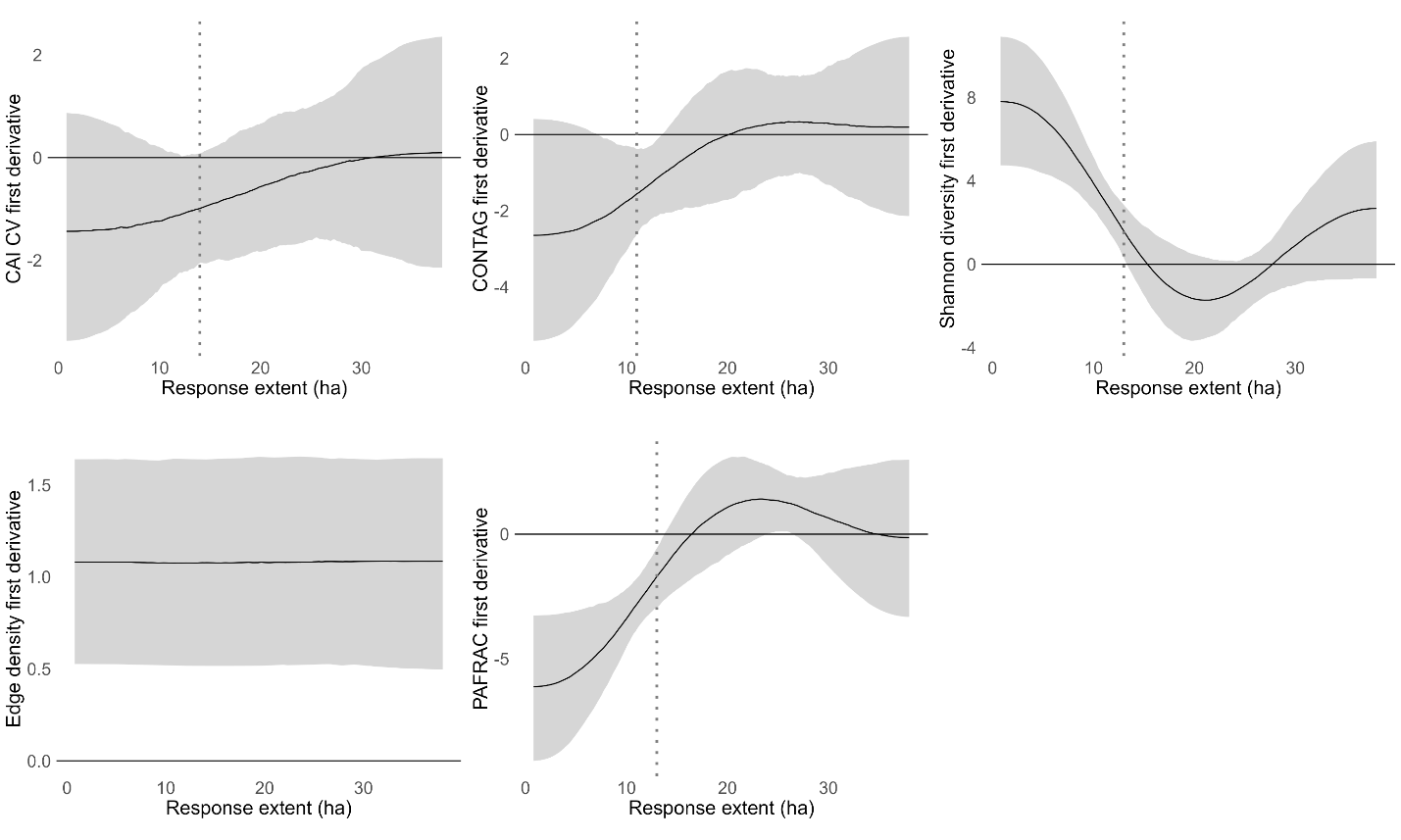


**Fig. S5** First derivatives of hierarchical generalized additive model (HGAM) smooths showing rate of change in predictor extent relative to response extent for five landscape configuration metrics. The first derivative represents the slope of the relationship between predictor extent and response extent at each point along the response extent gradient. Shaded regions indicate 95% confidence intervals. The horizontal dashed line at zero marks where the slope is flat (no change in predictor extent with response extent). Domain boundaries occur where derivatives transition (indicated by vertical dotted line) between significantly positive (CI above zero), non-significant (CI overlapping zero), or significantly negative (CI below zero).


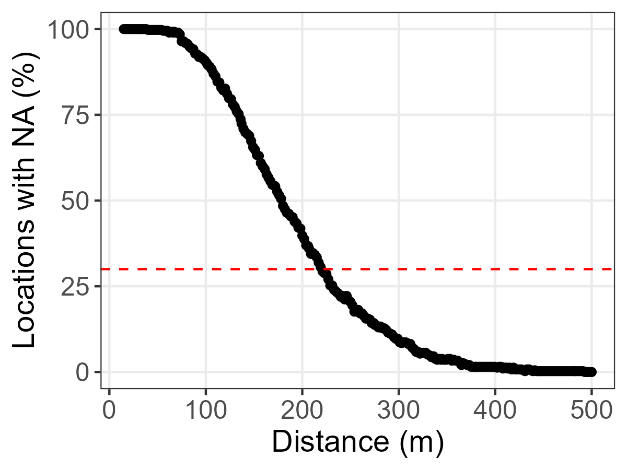


**Fig. S6.** Percentage of study locations (n = 392) with incalculable perimeter-area fractal dimension (PAFRAC) values across predictor extents. PAFRAC requires a minimum of 10 patches (Hesselbarth et al. 2019); sites with fewer patches at a given extent return NA values. The dashed red line indicates the 60% site availability threshold below which models are fit on a non-random subset of the most fragmented sites. This threshold is marked on PAFRAC panels in Figs. 2 and 4.
