# Supplementary figures and images for: Configurational heterogeneity drives songbird diversity at distinct spatial scales in managed boreal forests
Source: Landsc Ecol. 2026 Mar 27;41(5):86. doi: 10.1007/s10980-026-02341-y (PMC13156193; doi:10.1007/s10980-026-02341-y)

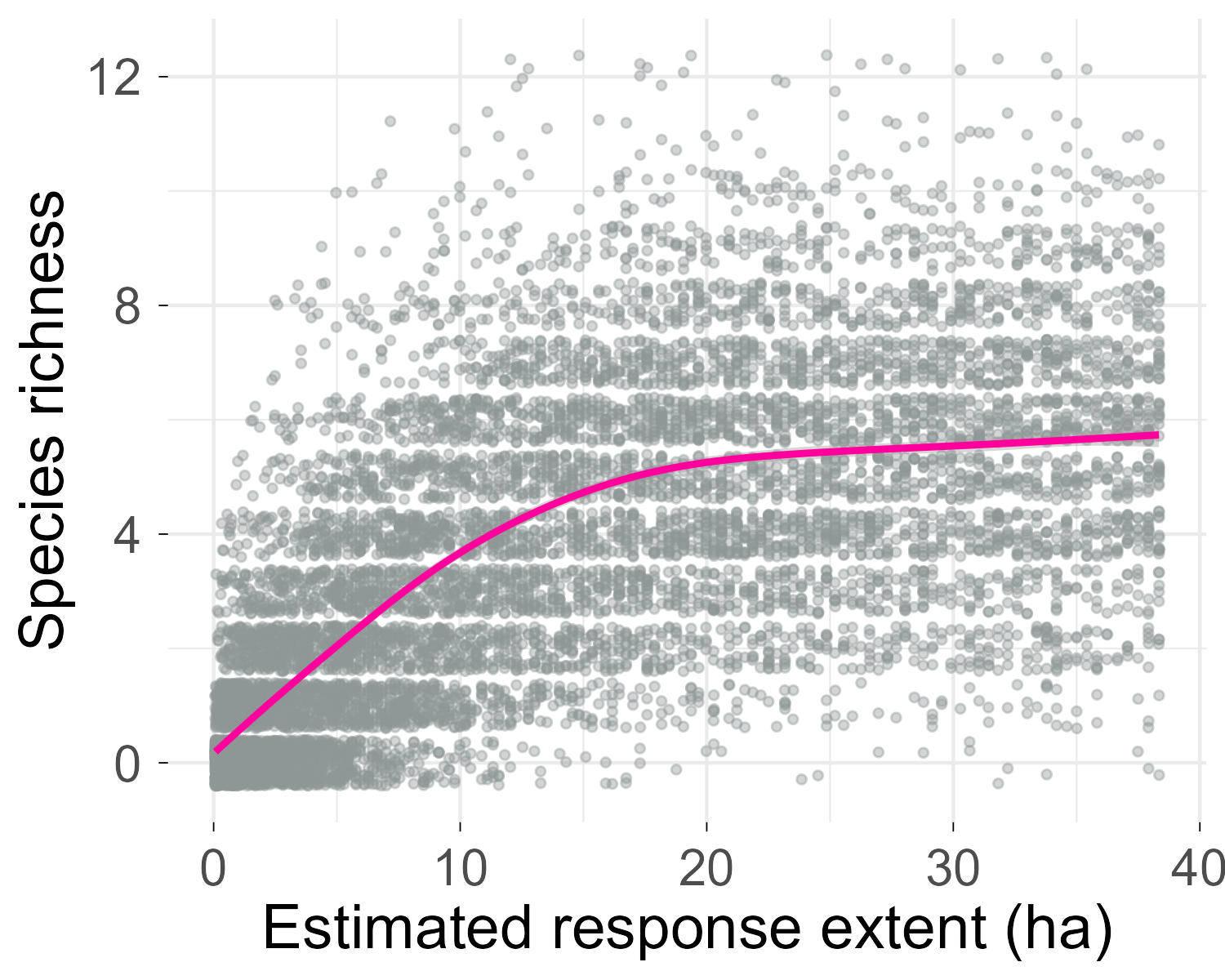

Supplement: Supplementary file 2 — Supplementary file2 (JPEG 526 KB) [file 10980_2026_2341_MOESM2_ESM.jpeg]

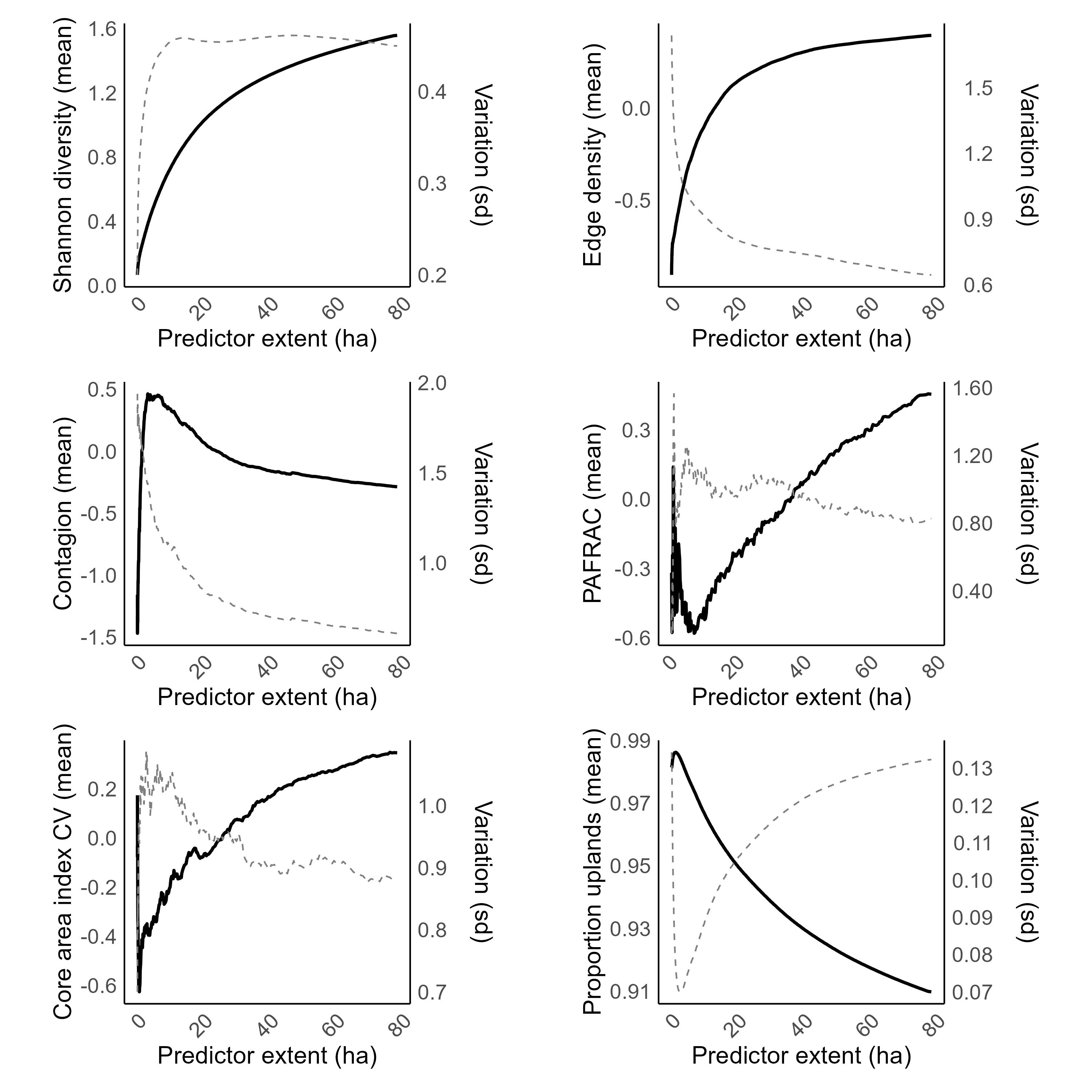

Supplement: Supplementary file 3 — Supplementary file3 (JPEG 1292 KB) [file 10980_2026_2341_MOESM3_ESM.jpeg]

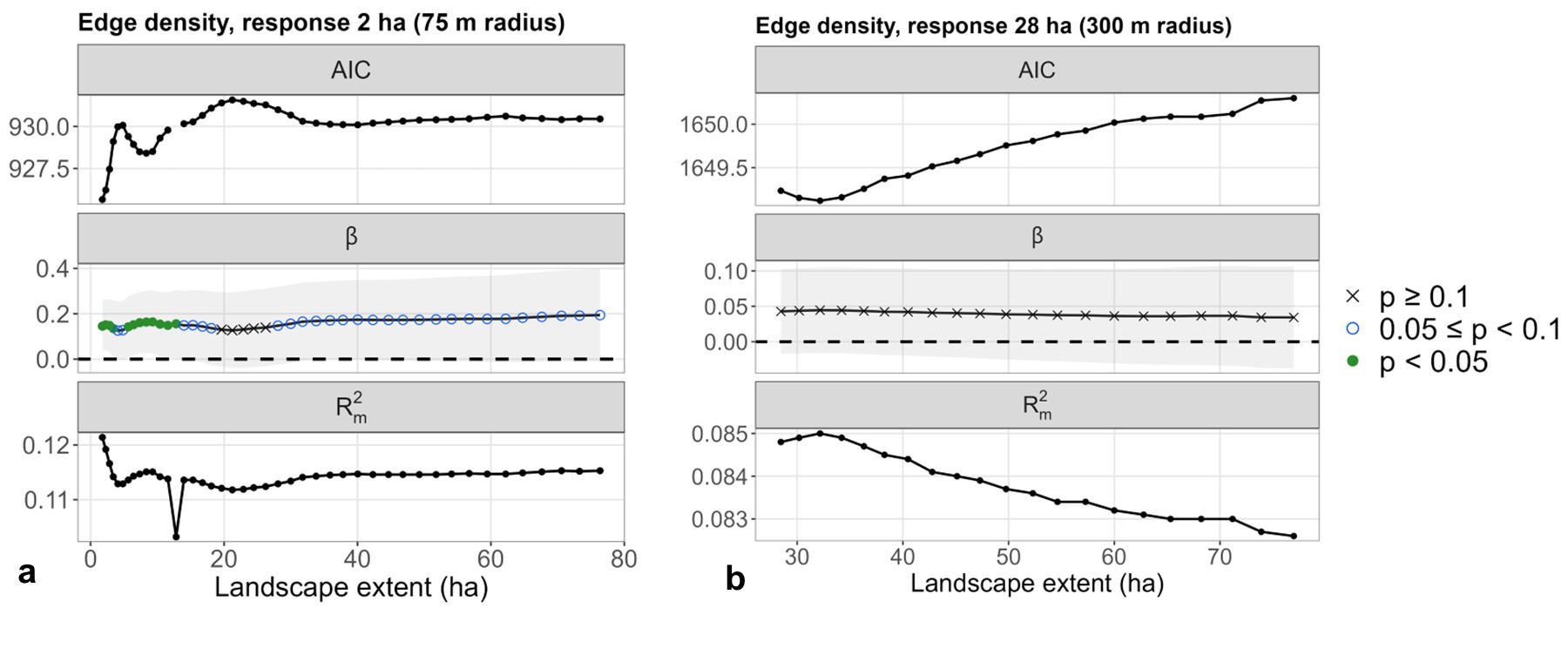

Supplement: Supplementary file 4 — Supplementary file4 (JPG 154 KB) [file 10980_2026_2341_MOESM4_ESM.jpg]

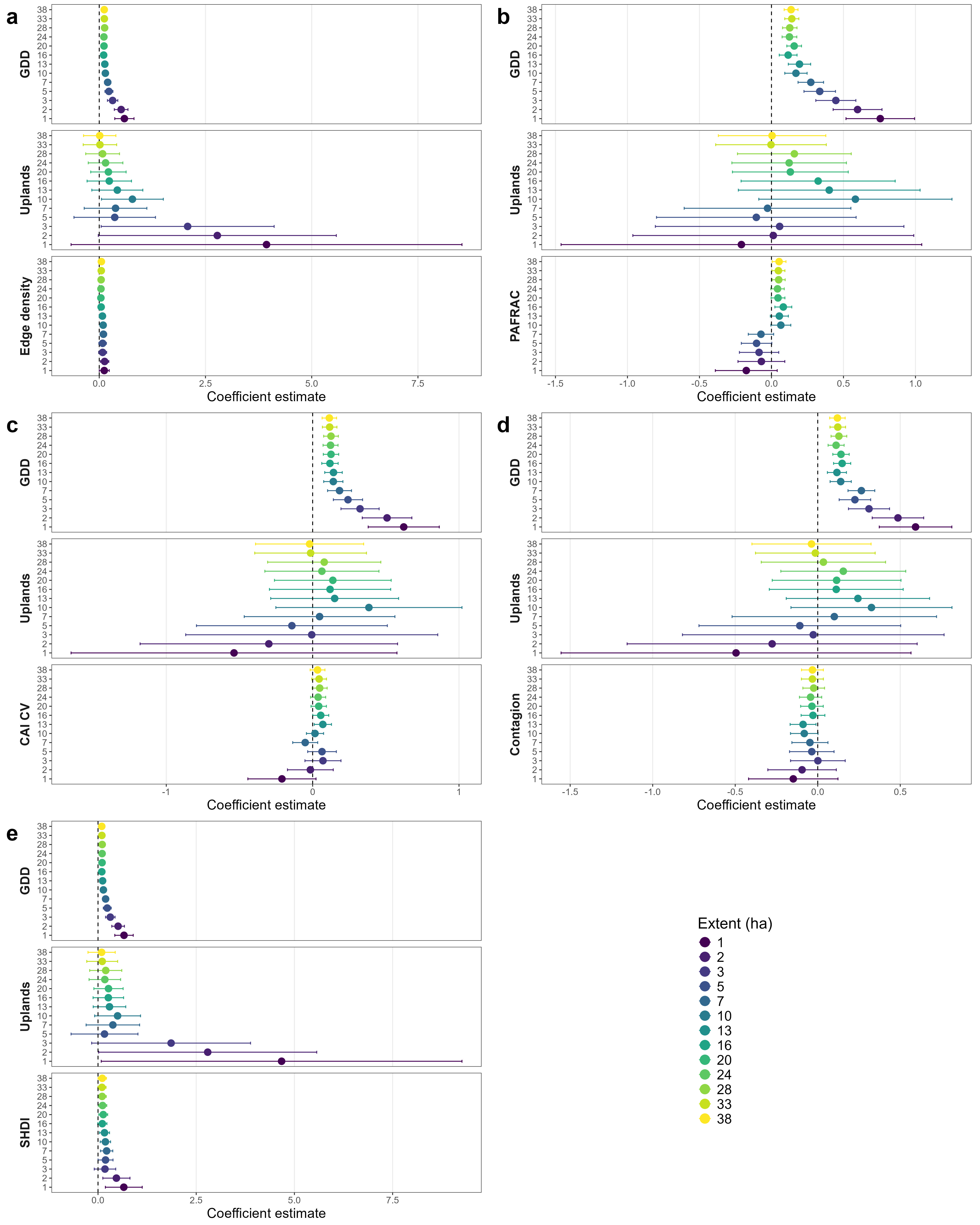

Supplement: Supplementary file 5 — Supplementary file5 (JPEG 3000 KB) [file 10980_2026_2341_MOESM5_ESM.jpeg]

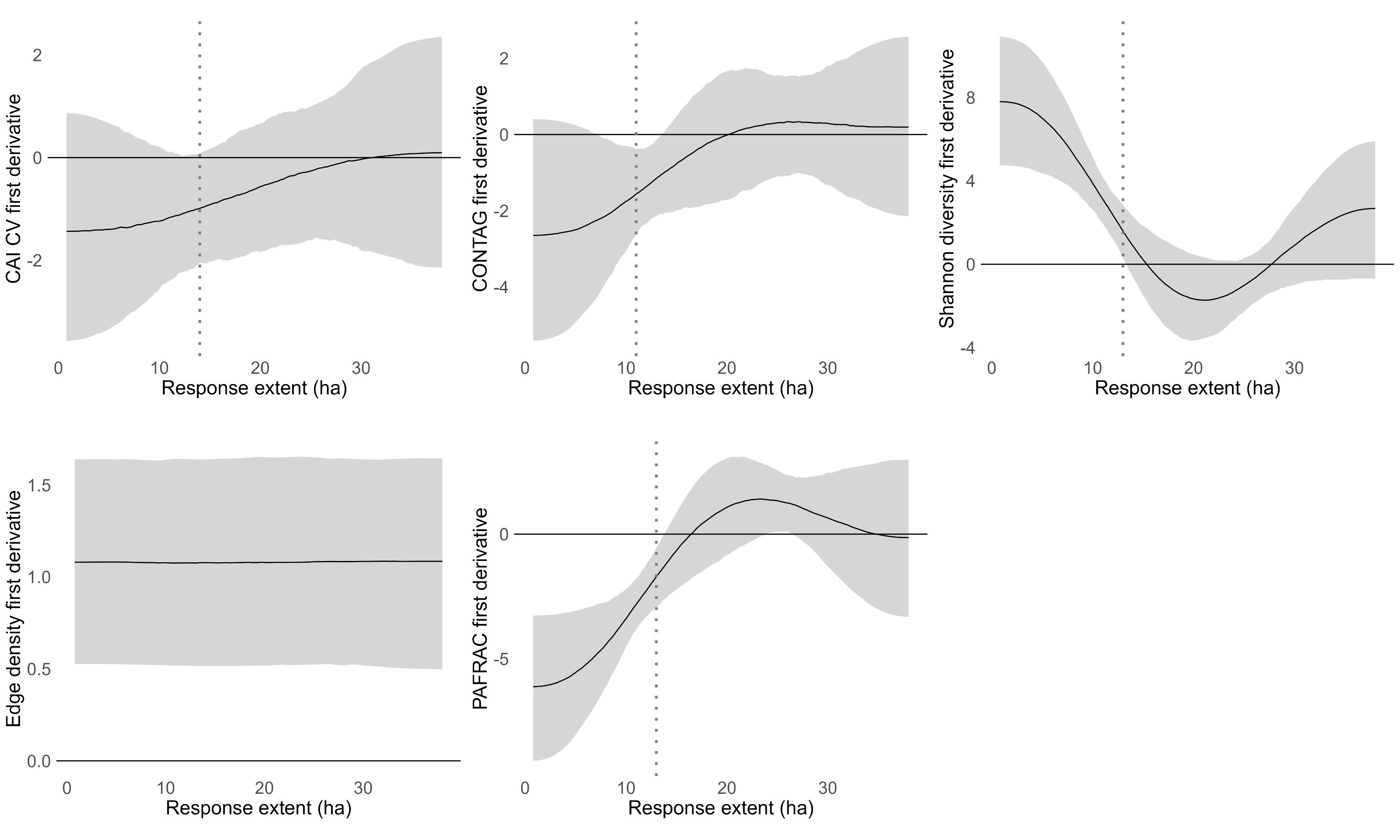

Supplement: Supplementary file 6 — Supplementary file6 (JPEG 555 KB) [file 10980_2026_2341_MOESM6_ESM.jpeg]

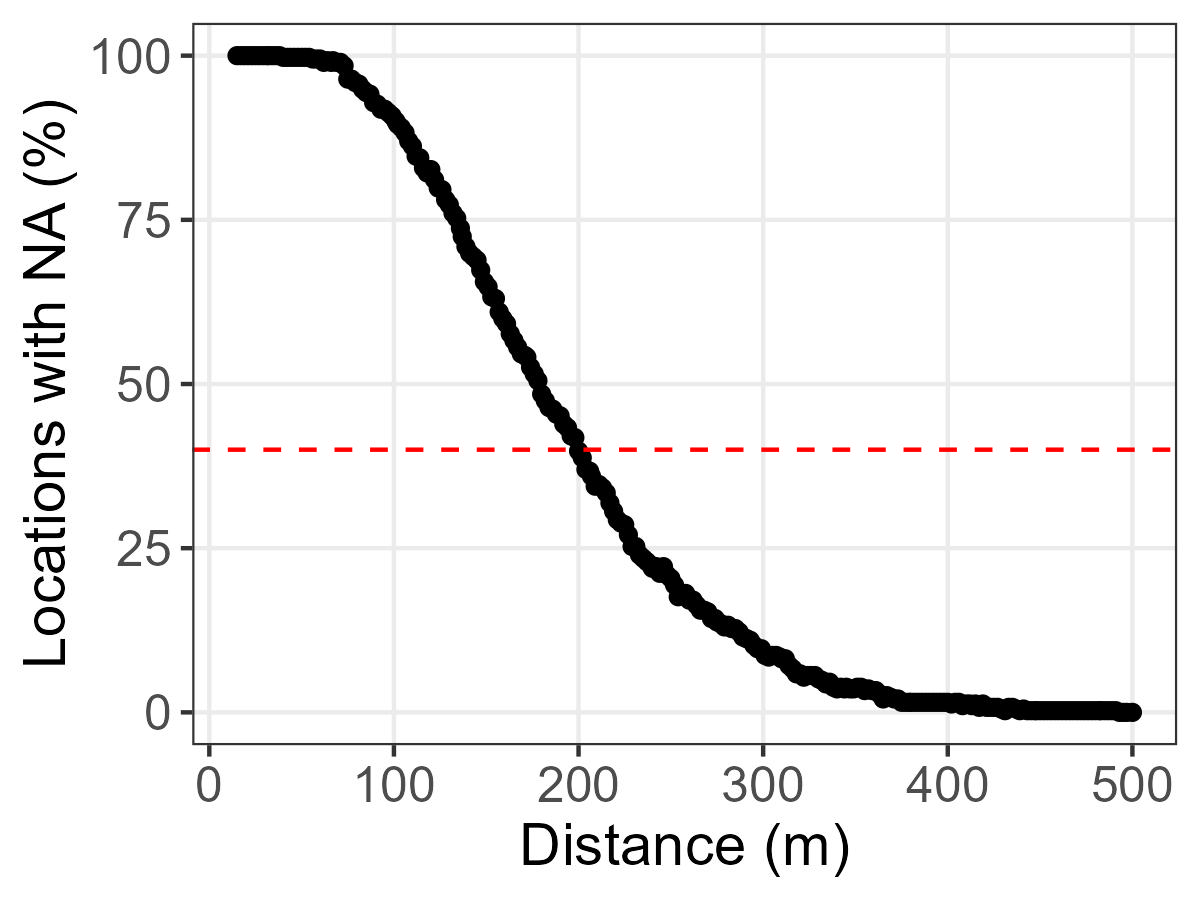

Supplement: Supplementary file 7 — Supplementary file7 (JPEG 194 KB) [file 10980_2026_2341_MOESM7_ESM.jpeg]
