# Supplementary material for: Configurational heterogeneity drives songbird diversity at distinct spatial scales in managed boreal forests
Source: Landsc Ecol. 2026 Mar 27;41(5):86. doi: 10.1007/s10980-026-02341-y (PMC13156193; doi:10.1007/s10980-026-02341-y)
Supplement: Supplementary file 8 — Supplementary file8 (DOCX 24 KB) [file 10980_2026_2341_MOESM8_ESM.docx]

| Common name | Scientific name | Order | Count |
| --- | --- | --- | --- |
| Alder Flycatcher | *Empidonax Alnorum* | Passeriformes | 146 |
| American Crow | *Corvus Brachyrhynchos* | Passeriformes | 7 |
| American Goldfinch | *Spinus Tristis* | Passeriformes | 3 |
| American Kestrel | *Falco Sparverius* | Falconiformes | 1 |
| American Redstart | *Setophaga Ruticilla* | Passeriformes | 12 |
| American Robin | *Turdus Migratorius* | Passeriformes | 136 |
| American Three-toed Woodpecker | *Picoides Dorsalis* | Piciformes | 2 |
| American Wigeon | *Mareca american* | Anseriformes | 1 |
| Baltimore Oriole | *Icterus Galbula* | Passeriformes | 1 |
| Bay-breasted Warbler | *Setophaga Castanea* | Passeriformes | 1 |
| Black and white Warbler | *Mniotilta Varia* | Passeriformes | 15 |
| Black-billed Magpie | *Pica Hudsonia* | Passeriformes | 1 |
| Black-capped Chickadee | *Poecile Atricapillus* | Passeriformes | 11 |
| Black-throated Green Warbler | *Setophaga Virens* | Passeriformes | 1 |
| Blue Jay | *Cyanocitta Cristata* | Passeriformes | 3 |
| Blue-headed Vireo | *Vireo Solitarius* | Passeriformes | 6 |
| Boreal Chickadee | *Poecile Hudsonicus* | Passeriformes | 3 |
| Brown Creeper | *Certhia Americana* | Passeriformes | 1 |
| Brown-headed Cowbird | *Molothrus Ater* | Passeriformes | 24 |
| Canada Goose | *Branta Canadensis* | Anseriformes | 4 |
| Canada Jay | *Perisoreus Canadensis* | Passeriformes | 63 |
| Canada Warbler | *Cardellina Canadensis* | Passeriformes | 1 |
| Cassin's Vireo | *Vireo Cassinii* | Passeriformes | 1 |
| Cedar Waxwing | *Bombycilla Cedrorum* | Passeriformes | 25 |
| Chipping Sparrow | *Spizella Passerina* | Passeriformes | 120 |
| Clay-colored Sparrow | *Spizella Pallida* | Passeriformes | 44 |
| Common Loon | *Gavia Immer* | Gaviiformes | 3 |
| Common Raven | *Corvus Corax* | Passeriformes | 17 |
| Common Redpoll | *Acanthis Flammea* | Passeriformes | 2 |
| Common Yellowthroat | *Geothlypis Trichas* | Passeriformes | 38 |
| Connecticut Warbler | *Oporornis Agilis* | Passeriformes | 2 |
| Dark-eyed Junco | *Junco Hyemalis* | Passeriformes | 98 |
| Downy Woodpecker | *Picoides pubescens* | Piciformes | 3 |
| Dusky Flycatcher | *Empidonax Oberholseri* | Passeriformes | 7 |
| European Starling | *Sturnus Vulgaris* | Passeriformes | 1 |
| Evening Grosbeak | *Coccothraustes Vespertinus* | Passeriformes | 1 |
| Fox Sparrow | *Passerella Iliaca* | Passeriformes | 2 |
| Golden-crowned Kinglet | *Regulus Satrapa* | Passeriformes | 7 |
| Greater Yellowlegs | *Tringa Melanoleuca* | Charadriiformes | 3 |
| Hairy Woodpecker | *Dryobates Villosus* | Piciformes | 4 |
| Hermit Thrush | *Catharus Guttatus* | Passeriformes | 72 |
| House Wren | *Troglodytes Aedon* | Passeriformes | 5 |
| Least Flycatcher | *Empidonax Minimus* | Passeriformes | 24 |
| LeConte's Sparrow | *Ammospiza Leconteii* | Passeriformes | 3 |
| Lesser Yellowlegs | *Tringa Flavipes* | Charadriiformes | 7 |
| Lincoln's Sparrow | *Melospiza Lincolnii* | Passeriformes | 133 |
| Magnolia Warbler | *Setophaga Magnolia* | Passeriformes | 35 |
| Mountain Chickadee | *Poecile Gambeli* | Passeriformes | 4 |
| Mourning Warbler | *Geothlypis Philadelphia* | Passeriformes | 36 |
| Nashville Warbler | *Leiothylpis Ruficapilla* | Passeriformes | 2 |
| Northern Flicker | *Colaptes Auratus* | Piciformes | 14 |
| Northern Waterthrush | *Parkesia Noveboracensis* | Passeriformes | 10 |
| Olive-sided Flycatcher | *Contopus Cooperi* | Passeriformes | 20 |
| Orange-crowned Warbler | *Leiothlypis Celata* | Passeriformes | 104 |
| Ovenbird | *Seiurus Aurocapilla* | Passeriformes | 37 |
| Pacific Wren | *Troglodytes Pacificus* | Passeriformes | 1 |
| Palm Warbler | *Setophaga Palmarum* | Passeriformes | 25 |
| Pileated Woodpecker | *Dryocopus Pileatus* | Piciformes | 5 |
| Pine Siskin | *Spinus Pinus* | Passeriformes | 62 |
| Purple Finch | *Haemorhous Purpureus* | Passeriformes | 2 |
| Red Crossbill | *Loxia Curvirostra* | Passeriformes | 2 |
| Red-breasted Nuthatch | *Sitta Canadensis* | Passeriformes | 27 |
| Red-eyed Vireo | *Vireo Olivaceus* | Passeriformes | 98 |
| Red-winged Blackbird | *Agelaius Phoeniceus* | Passeriformes | 2 |
| Rose-breasted Grosbeak | *Pheucticus Ludovicianus* | Passeriformes | 38 |
| Ruby-crowned Kinglet | *Regulus calendula* | Passeriformes | 47 |
| Ruffed Grouse | *Bonasa Umbellus* | Galliformes | 10 |
| Savannah Sparrow | *Passerculus Sandwichensis* | Passeriformes | 1 |
| Say's Phoebe | *Sayornis saya* | Passeriformes | 1 |
| Solitary Sandpiper | *Tringa Solitaria* | Charadriiformes | 2 |
| Song Sparrow | *Melospiza Melodia* | Passeriformes | 2 |
| Swainson's Thrush | *Catharus Ustulatus* | Passeriformes | 177 |
| Swamp Sparrow | *Melospiza Georgiana* | Passeriformes | 4 |
| Tennessee Warbler | *Leiothlypis Peregrina* | Passeriformes | 140 |
| Townsend's Solitaire | *Myadestes Townsendi* | Passeriformes | 9 |
| Varied Thrush | *Ixoreus Naevius* | Passeriformes | 40 |
| Vesper Sparrow | *Pooecetes Gramineus* | Passeriformes | 4 |
| Warbling Vireo | *Vireo Gilvus* | Passeriformes | 88 |
| Western Flycatcher | *Empidonax Difficilis* | Passeriformes | 1 |
| Western Tanager | *Piranga Ludoviciana* | Passeriformes | 18 |
| Western Wood Pewee | *Contopus Sordidulus* | Passeriformes | 11 |
| White-breasted Nuthatch | *Sitta Carolinensis* | Passeriformes | 2 |
| White-crowned Sparrow | *Zonotrichia Leucophrys* | Passeriformes | 29 |
| White-throated Sparrow | *Zonotrichia Albicollis* | Passeriformes | 274 |
| White-winged Crossbill | *Loxia Leucoptera* | Passeriformes | 20 |
| Wilson's Snipe | *Gallinago Delicata* | Charadriiformes | 65 |
| Wilson's Warbler | *Cardellina Pusilla* | Passeriformes | 12 |
| Winter Wren | *Troglodytes Hiemalis* | Passeriformes | 10 |
| Yellow Warbler | *Setophaga Petechia* | Passeriformes | 15 |
| Yellow-bellied Flycatcher | *Empidonax Flaviventris* | Passeriformes | 15 |
| Yellow-bellied Sapsucker | *Sphyrapicus Varius* | Piciformes | 3 |
| Yellow-rumped Warbler | *Setophaga Coronata* | Passeriformes | 129 |

Table S1. Detected species (92), their order, and number of sites in which they were detected, from a total of 392 sites. Only Passeriformes were included in the analysis.
